# Heterogeneous changes in electricity consumption patterns of residential distributed solar consumers due to battery storage adoption

**DOI:** 10.1016/j.isci.2022.104352

**Published:** 2022-05-04

**Authors:** Yueming (Lucy) Qiu, Bo Xing, Anand Patwardhan, Nathan Hultman, Huiming Zhang

**Affiliations:** 1 School of Public Policy, University of Maryland, College Park, MD 20742, USA; 2 Department of Forecasting, Research & Economic Development, Salt River Project, Tempe, AZ 85281, USA; 3 China Institute of Manufacturing Development, Nanjing University of Information Science & Technology, Nanjing, Jiangsu Province 210044, China

**Keywords:** energy policy, Electrical system, energy management, energy Modeling, energy storage, energy systems

## Abstract

This study provides an empirical assessment of how adopting battery storage units can change the electricity consumption patterns of PV consumers using individual-consumer-level hourly smart meter data in Arizona, United States. We find that on average after adding batteries, PV consumers use more solar electricity to power their houses and send less solar electricity back to the grid. In addition, adding battery storage reduces electricity needed from the grid during system peak hours, helping utilities better flatten the load curves. Most importantly, we find a large degree of heterogeneity in the changes in electricity consumption patterns due to adopting battery storage that are not consistent with engineering or economic principles such as those not maximizing consumers’ economic benefits. Such heterogeneous changes imply that utilities and policymakers need to further study the underlying behavioral reasons in order to maximize the social benefits of battery storage and PV co-adoption.

## Highlights

• Adding battery storage changes solar consumers’ electricity consumption patterns
• Adding battery increases self-consumption of solar electricity
• Battery reduces electricity needed from the grid during system peak hours
• We find a large degree of heterogeneity in the changes in consumption patterns

## Graphical abstract

**Figure:**
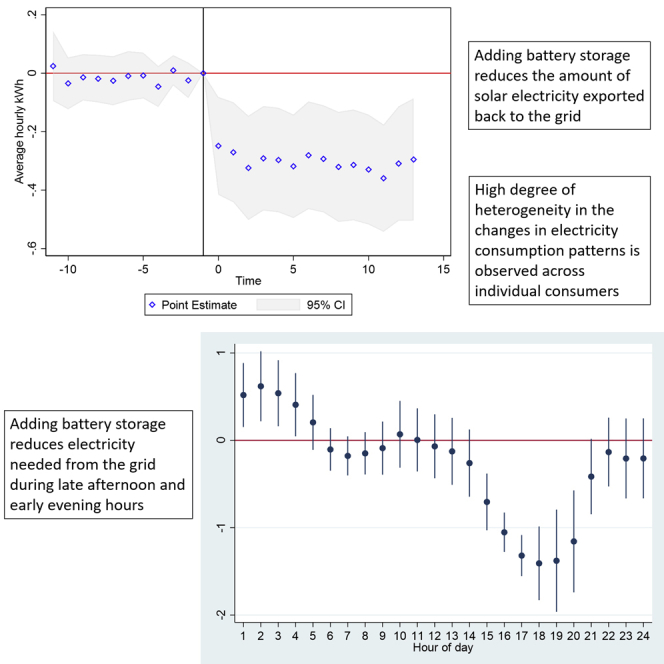

## Introduction

Residential battery storage for households has gained a great deal of attention in the recent few years (Agnew and Dargusch, 2015). The annual installations of residential battery storage, although still relatively slow among all households, have jumped by 81 times from 2014 to 2018 in the U.S. (EIA, 2018). Residential installations even exceeded utility-scale installations in 2018. Such storage systems offer consumers the benefits of power reliability and resilience, and bill savings (i.e. by charging the battery at lower electricity price hours and discharging at higher price hours). For utilities, residential storage systems can potentially help flatten the load curves and provide grid support services such as ancillary services and demand response. Battery storage has been a hot topic in recent academic literature, but mostly in engineering (Ratnam et al., 2015a), economics simulation (Dorsey et al., 2020; Jargstorf et al., 2015), and techno-economics studies (Ranaweera and Midtgård, 2016; Stephan et al., 2016).

Combining battery storage with solar photovoltaics (PVs) has the potential to significantly enhance the degree of decarbonization, the resilience of the power systems, and the private benefits of technology adopters (Freitas Gomes et al., 2020; Nyholm et al., 2016). Without batteries, solar PV consumers need to export excess solar electricity back to the grid if the amount of electricity needed at the house is smaller than that generated by the solar panels. With batteries, PV consumers can store solar electricity in the battery, and then the battery can discharge at hours when there is not enough solar electricity such as in the evening hours, thus increasing the self-consumption of solar electricity.

Government incentives such as tax credits and rebates widely exist to encourage consumers to adopt batteries and PVs and some consumers indeed adopt both technologies. Despite the low current rate of PV and battery co-adoption, a growing number of consumers are co-adopting these technologies according to the data from Tracking the Sun (Barbose et al., 2021). Large penetration of PV and battery co-adopters can also be potentially disruptive and impose challenges to the electric grid management such as balancing the supply and demand of the power grid with the presence of decentralized and less predictable consumer electricity importing and exporting behaviors (Appen et al., 2013; Tran et al., 2019). An understanding of how electricity demand is affected by PV and battery co-adoption is central to determining the grid-level and consumer-level (private and external) benefits and costs of co-adoption that may justify government intervention and bear directly on resource planning of utilities and utility regulators (Hill et al., 2012).

An empirical analysis of the behavior changes due to battery adoption of PV consumers is needed for the following reasons. Current models to understand the electricity consumption behaviors of co-adopters of PV and battery have one major limitation—i.e., these models are largely based on engineering, economics simulation, and techno-economics studies (Qiu et al., 2019; Qiu et al., 2021) and do not account for actual consumer behaviors and the related heterogeneity. However, existing studies have found that consumers’ actual behaviors can deviate from engineering and economic predictions (Fowlie et al., 2018; Scheer et al., 2013; Zivin and Novan, 2016; Qiu and Kahn, 2019), and such deviations will be heterogeneous (Liang et al., 2017) depending on consumer characteristics. For example, some studies show that residential solar consumers increase their overall electricity consumption because of “free” solar power—the so-called rebound effects (Qiu et al., 2019; Spiller et al., 2017). Consumer behaviors can also deviate from rational economic choices (Gowdy, 2008; Maréchal, 2010). In this paper, we show that battery charging profiles are not maximizing adopter benefits for some consumers on time-of-use (TOU) pricing because the batteries discharge in some off-peak hours when electricity prices are lower; instead, batteries should discharge more during the peak hours to avoid buying more expensive electricity from the grid. As another example, some consumers not only reduce electricity delivered during peak hours but also during off-peak hours.

Previous studies that examine the impact of residential co-adoption of PV and battery have used simulation approaches or relied on very small-scale experimental demonstrations (Khiareddine et al., 2015; Ratnam et al., 2015b; Teymour et al., 2014; Zhong et al., 2019). Simulation-based studies in engineering literature have explored the integration of these technologies into the electricity grid (Ding et al., 2010; Li et al., 2018; O’Shaughnessy et al., 2018). While engineering models can predict these impacts based on solutions to optimization problems and other simulation methods, they necessarily make assumptions about how people interact with the technologies and the electricity tariffs they face (Muratori, 2018). Simulation-based studies often have important common limitations as discussed in Muratori (2018): (a) assuming that technologies are used in a certain way (e.g., EVs charged mostly in off-peak hours) and (b) using an average and pre-determined technology-using profile (such as charging profiles) for all consumers in the models, or (c) assuming certain categories or distributions of behavioral profiles that do not reflect the actual heterogeneous behaviors of consumers (Dong et al., 2018).

Existing relevant empirical studies only examine the changes due to single-technology adoption and its impact on electricity demand and the electric grid; no empirical studies examine electricity consumption changes of PV and battery co-adopters. For example, some empirical studies have examined the electricity consumption behaviors of PV owners (Qiu et al., 2019; Deng and Newton, 2017; McKenna et al., 2018; La Nauze, 2019; Spiller et al., 2017) but they do not look at the co-adoption of other technologies. Despite that the impacts of individual technologies are empirically analyzed, the individual technology’s impacts are not additive in a co-adoption system because consumers will change how they utilize one technology when they add another technology.

This project fills these major gaps by providing an empirical assessment of the heterogeneous changes in electricity consumption patterns due to battery adoption of PV consumers. In this study, we use the individual-consumer-level hourly smart meter data from 2013 to 2020 of battery and PV co-adopters and PV-only consumers to estimate the changes in hourly electricity delivered to the consumers and hourly electricity exported back to the grid by the consumers due to adding battery storage to homes with PV. We use difference-in-differences and matching methods, plus group-specific time trends, to correct for potential endogeneity issues. There are several important findings. First, our results show that on average, after adding batteries, PV consumers use more solar electricity to power their houses and send less solar electricity back to the grid, consistent with a widely held hypothesis established through modeling (Castillo-Cagigal et al., 2011; Yu, 2021). Second, adding battery storage reduces electricity needed from the grid during peak hours such as late afternoon and early evening in the summer, helping utilities better flatten the load curves and ensure grid stability. Third, we find a large degree of heterogeneity in the changes in electricity consumption patterns due to adopting battery storage. For example, some battery charging profiles are maximizing consumer economic benefits while others are not. Such heterogeneous changes imply that utilities and policymakers need to further study the underlying behavioral reasons in order to maximize the social benefits of battery storage and PV co-adoption. When these underlying behaviors are further analyzed and confirmed in future studies, utilities and policymakers may be able to take a more targeted approach to deal with potential consumer behavioral issues such as inattention, information asymmetry, and cognitive constraint.

## Results

Our data come from a major utility company called Salt River Project (SRP) which serves the Phoenix metropolitan area in Arizona. In our final analysis sample, there are 56 PV consumers who added battery storage units at a later time and these consumers form our treatment group. 1378 non-battery PV consumers form the control group. Out of the 56 battery consumers, five consumers are on a price plan called E21 time-of-use rate (TOU), 12 consumers are on E23 increasing block rate (non-TOU rate), 12 consumers are on E26 TOU rate, and 24 are on E27 TOU rate. The remaining three consumers are on three other plans. Details of these price plans, with the hours of peak and off-peak periods and marginal prices for each hour, can be found in Table S2 in the supplementary information. We use a two-way fixed effects panel regression model to estimate the impact of battery adoption on electricity consumption patterns. Details of the models and data can be found in STAR Methods.

### Event study results

**Figure 1:**
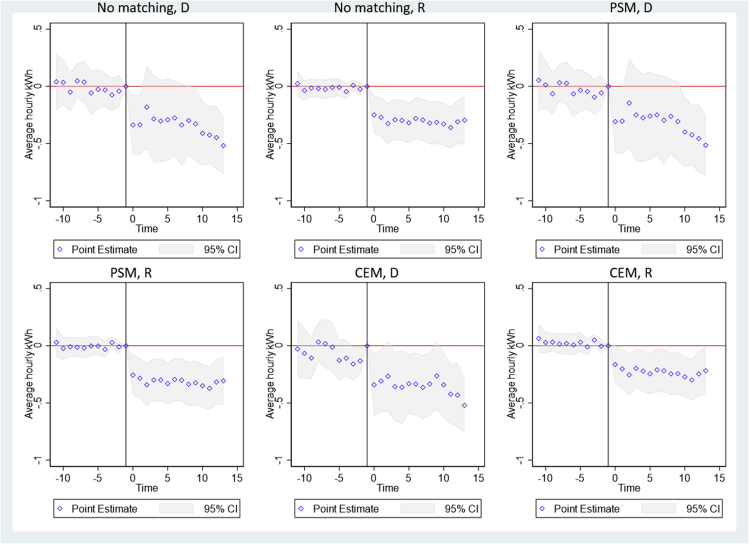
Event study analysis results y axis indicates the difference in hourly electricity delivered (purchases) or received (exports) between the treatment and the control consumers. The dots indicate point estimates and the shaded areas indicate 95% confidence intervals. Weeks prior to the battery adoption are indicated by −10 to 0 in the X axis, and weeks after are indicated by 0–15. The difference is normalized to be zero at time −1. “D” indicates electricity delivered from the grid to the consumers; “R” indicates electricity received by the grid from the consumers. “No matching” indicates using the randomly selected non-battery PV consumers as the control group; “PSM” indicates event study regressions weighted by the weights assigned in the propensity score matching process; “CEM” indicates event study regressions weighted by the weights assigned in the coarsened exact matching process.

We first run an event study (see STAR Methods) to check for the parallel trend assumption. Figure 1 shows the results of the event study analysis for three sets of samples: without matching, propensity score matching (PSM), and coarsened exact matching (CEM). For both the electricity delivered to the consumers and the electricity received by the grid, in the pre-treatment period, the difference between the treatment and control groups is not statistically significant. This indicates that the treatment and control consumers share similar electricity using patterns prior to the adoption of batteries and thus confirms the parallel trend assumption for the DID analysis. In the post-treatment period, both the electricity delivered and electricity received decline. This indicates that after adding batteries, PV consumers use more solar electricity to power their houses and send less solar electricity back to the grid, consistent with a widely held hypothesis established through modeling.

### Overall effects on electricity delivered and received

**Figure 2:**
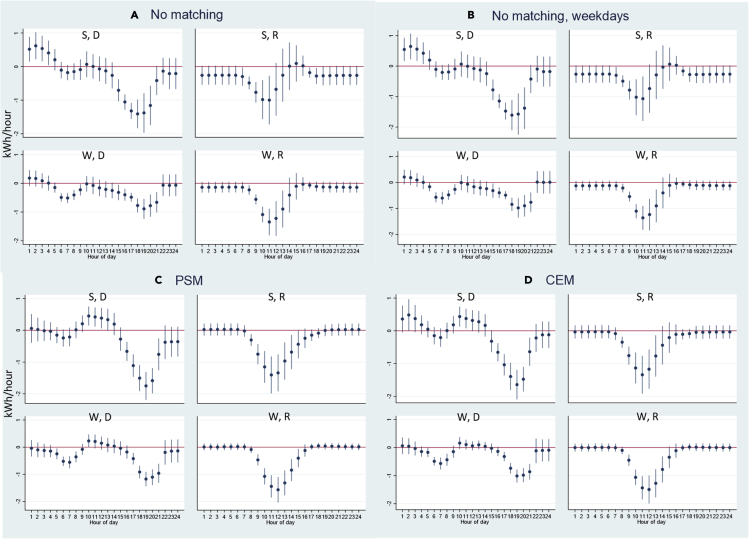
Impact of battery adoption on electricity delivered (purchases) or received (exports), overall sample y axis is the change in hourly electricity measured in kWh/hour due to battery adoption; x axis is the hour-of-day. The dots indicate point estimates and the vertical lines indicate 95% confidence intervals. “S” indicates summer months (May–Oct); “W” indicates winter months (Jan–Apr, Nov–Dec); “D” indicates electricity delivered from the grid to the consumers; “R” indicates electricity received by the grid from the consumers. Panel A is for “No matching”, which indicates using the randomly selected non-battery PV consumers as the control group; Panel B is for "No matching" with only weekdays; Panel C is for “PSM”, which indicates regressions weighted by the weights assigned in the propensity score matching process; Panel D is for “CEM”, which indicates regressions weighted by the weights assigned in the coarsened exact matching process. Detailed coefficients can be found in Table S4 in supplementary information.

Figure 2 shows the changes in electricity delivered (purchases) and received (exports) by season due to battery adoption based on the average profiles of all 56 battery consumers. First, comparing panel (a) and panel (b), the results including both weekdays and weekends (panel a) are very similar to the results including weekdays only (panel b). Thus, we focus our discussion on the results including both weekdays and weekends. Second, comparing among panels (a, c, & d), the results are generally consistent but since PSM generates a larger sample compared to CEM and PSM controls for the difference in the levels of electricity using patterns, we focus our discussion on the results from PSM.

From panel (c) PSM results, in the summer months, battery adoption enables PV consumers to reduce the electricity needed from the grid in the late afternoon and early evening hours, and reduces the amount of electricity exported back to the grid during the morning and early afternoon. This indicates that batteries allow the PV consumers to store solar electricity during the day, and batteries discharge to power the house in the late afternoon and early evening hours, during which electricity demand is usually the highest for residential consumers. In addition, the PV plus battery consumers increase their electricity delivered from the grid from 10 a.m. to 1 p.m., indicating that in some households, batteries might be also charging using grid electricity, in addition to the solar electricity during these hours. As will be discussed later, this increase in electricity in the late morning hours could be due to lower electricity prices during those hours so that batteries can charge using cheaper electricity. In the winter months, battery adoption enables PV consumers to reduce the electricity needed from the grid in both early morning hours (with increased electric heating needs) and the late afternoon and early evening hours, which are the two typical peak hour windows in the winter months for residential electricity usage. The electricity exported back to the grid in the winter months also decreases during the day.

Overall, the results indicate that battery storage enables consumers to use more solar electricity during hours when there is not enough solar electricity to power the whole house, such as in the late afternoon and early evening hours in summer and winter (and in the early morning hours in the winter) and thus reduce their electricity needed from the grid during these peak demand hours. Such reduction is also helpful for utilities to reduce the system load pressure during these peak hours so that the load curves can be better flattened and the grid stability can be better ensured. In the next section, we examine whether consumers’ behaviors are consistent with the incentives provided by the electricity pricing signals.

### Results by electricity price plans

**Figure 3:**
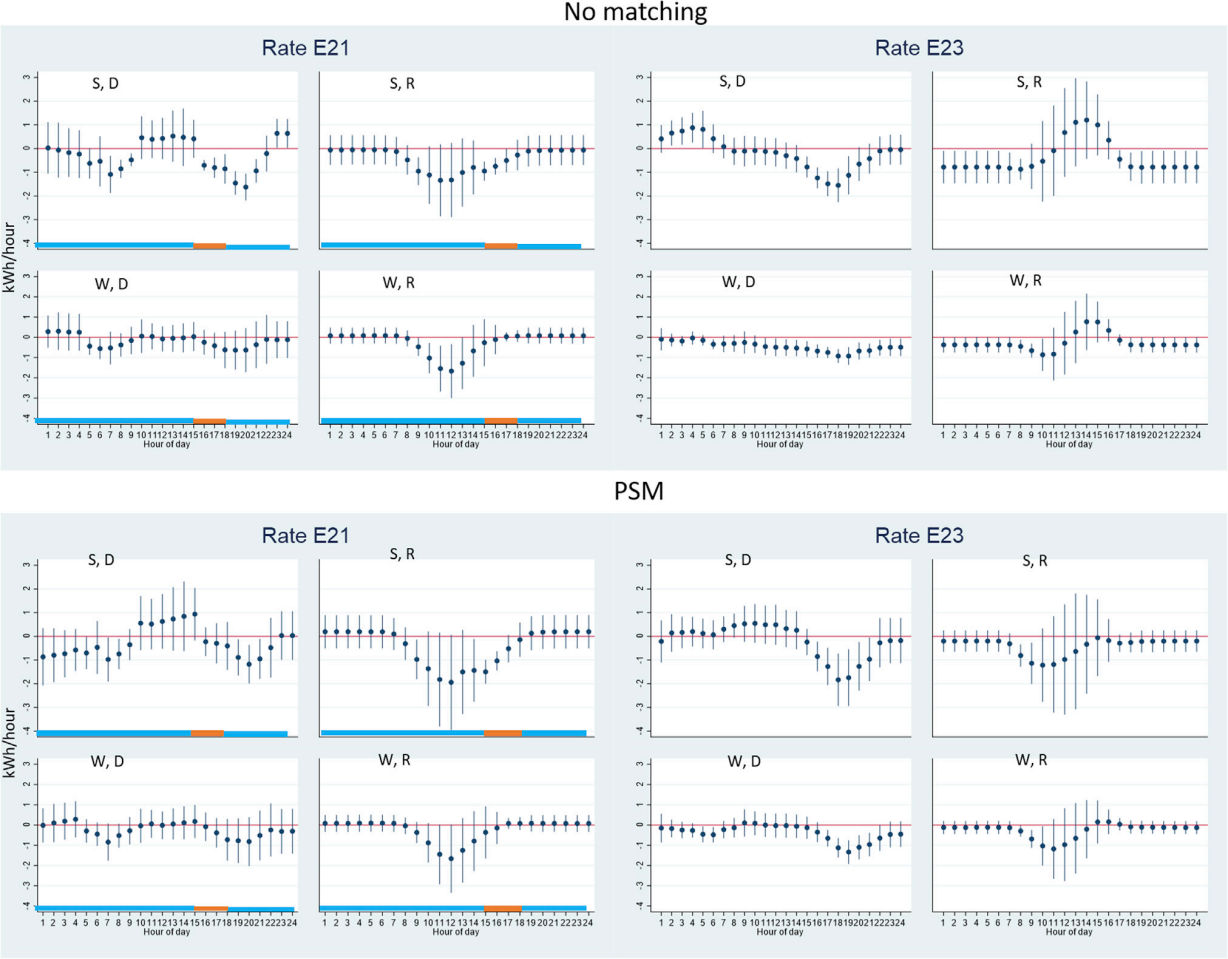
Impact of battery adoption on electricity delivered (purchases) or received (exports), rate plans E21 (5 consumers) and E23 (12 consumers) Orange color indicates peak hours with higher electricity prices; blue color indicates off-peak hours with lower electricity prices. y axis is the change in hourly electricity measured in kWh/hour due to battery adoption; x axis is the hour-of-day. The dots indicate point estimates and the vertical lines indicate 95% confidence intervals. “S” indicates summer months (May–Oct); “W” indicates winter months (Jan–Apr, Nov–Dec); “D” indicates electricity delivered from the grid to the consumers; “R” indicates electricity received by the grid from the consumers. “No matching” indicates using the randomly selected non-battery PV consumers as the control group; “PSM” indicates regressions weighted by the weights assigned in the propensity score matching process. Detailed coefficients can be found in Table S5 in supplementary information.

Figure 3 shows the consumers that are on E21 and E23 rates. We focus our discussion on the PSM results. E21 is a time-of-use rate with 3–6 p.m. as the peak hours with higher electricity prices and the rest off-peak hours with lower electricity prices. Note that in our sample there are only five consumers on E21 plan, thus caution is needed when generalizing the results to a broader population. In the summer months, E21 consumers on average exhibit patterns that are not fully consistent with standard economics principles. In the summer months, the electricity delivered to the consumers decreases from 7 to 9 p.m. and 7–8 a.m. during which electricity prices are lower. The electricity delivered does not reduce during 3–6 p.m. during which electricity prices are higher. E21 consumers reduce the electricity exported back to the grid from 9 a.m. to 5 p.m., among which 3–5 p.m. fall into the peak hours with higher electricity prices. This indicates that consumers are not fully responding to the TOU pricing signals. An E21 consumer that is fully maximizing the economic benefits from energy bill savings should increase the electricity exported back to the grid from 3 to 6 p.m. so that they can gain more solar electricity income, or use more battery electricity during 3–6 p.m. to power the house (in which case, we will see a decrease in electricity delivered during these hours) to avoid buying electricity at a higher cost. We see neither of these happen in the summer nor the winter, which deviates from what engineering or economic models predict. Future studies are needed to examine the underlying factors for such deviations such as inattention, lack of knowledge of the technology or pricing schedule, or cognitive constraints.

The E23 rate is a non-TOU rate with no intra-day variation in marginal electricity prices, which indicates that the opportunity cost of consuming grid and solar electricity is the same throughout the day. Thus, the timing of electricity usage changes does not have any implications for maximizing economic benefits. Figure 3 shows that adopting batteries cause E23 PV consumers to reduce electricity needed from the grid from 4 to 9 p.m. in both summer and winter and from 5 to 6 a.m. in the winter, and reduce electricity exported back to the grid from 8 to 9 a.m. in both summer and winter. While the non-TOU rate consumers cannot leverage the price differentials at different hours of day, the rationales for them to purchase a battery are to increase the self-consumption of solar electricity and to be able to have backup power in case of a blackout.

**Figure 4:**
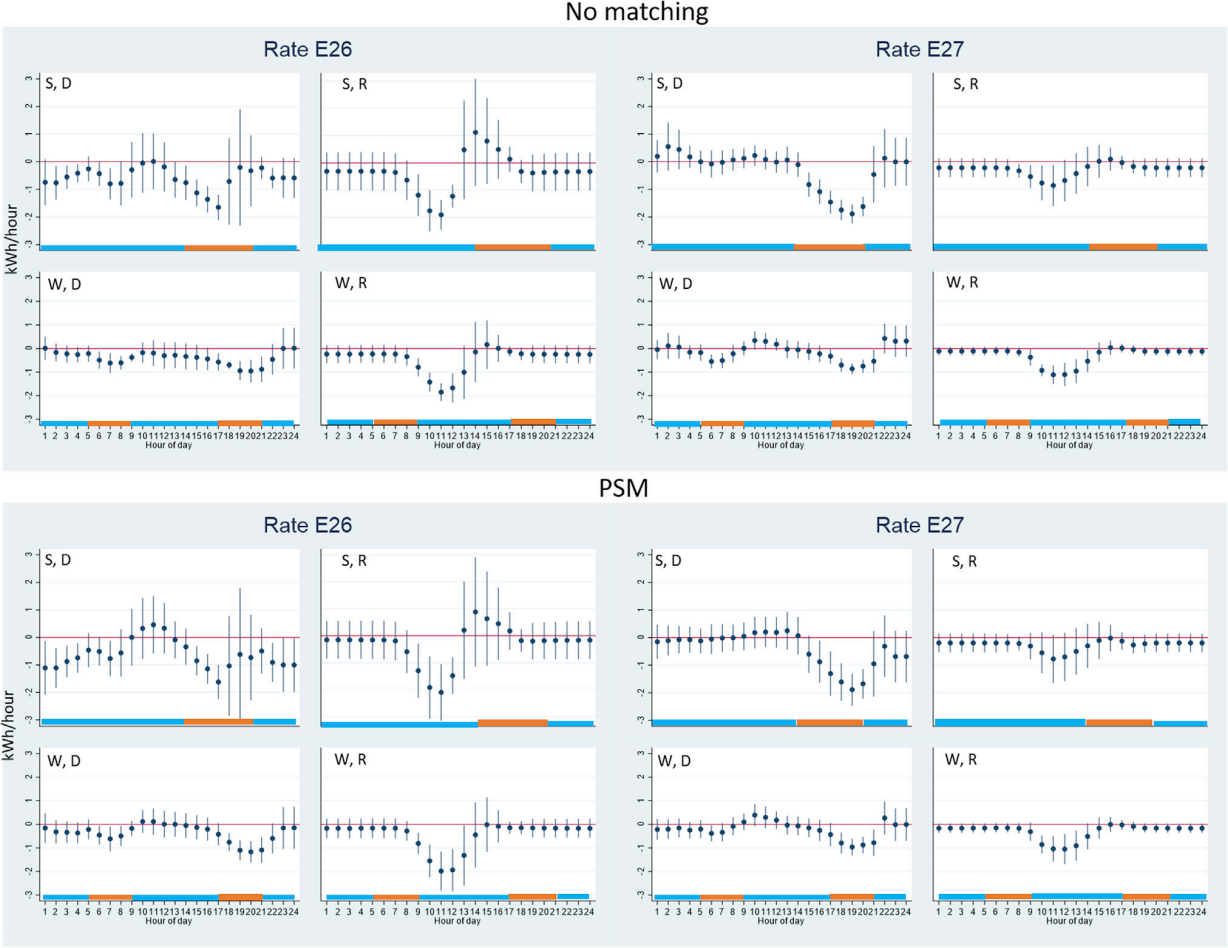
Impact of battery adoption on electricity delivered (purchases) or received (exports), rate plans E26 (12 consumers) and E27 (24 consumers) Orange color indicates peak hours with higher electricity prices; blue color indicates off-peak hours with lower electricity prices. y axis is the change in hourly electricity measured in kWh/hour due to battery adoption; x axis is the hour-of-day. The dots indicate point estimates and the vertical lines indicate 95% confidence intervals. “S” indicates summer months (May–Oct); “W” indicates winter months (Jan–Apr, Nov–Dec); “D” indicates electricity delivered from the grid to the consumers; “R” indicates electricity received by the grid from the consumers. “No matching” indicates using the randomly selected non-battery PV consumers as the control group; “PSM” indicates regressions weighted by the weights assigned in the propensity score matching process. Detailed coefficients can be found in Table S6 in supplementary information.

For both E26 and E27 rates, the summer peak hours are from 2 to 8 p.m. and winter peak hours are 5–9 a.m. and 5–9 p.m. A PV + battery consumer that fully maximizes economic benefits from bill savings should then reduce the electricity delivered during the peak hours, and/or increase electricity exported back to the grid during these peak hours. If battery capacity allows, a consumer can also increase electricity delivered during off-peak hours and then increase electricity exported back to the grid during peak hours. Figure 4 shows that the electricity consumption patterns of E26 and E27 consumers are generally consistent with the economic predictions (maximizing bill savings), with the exception of the summer patterns of E26 consumers. E27 consumers’ patterns are consistent with economic predictions by reducing electricity delivered during almost the entire peak hours: 2–8 p.m. in the summer and 5–9 a.m. and 5–9 p.m. in the winter. Please note that the electricity consumption changes we estimated are the changes compared to the behaviors before battery adoption. In other words, these TOU consumers could already have a reduced electricity demand during peak hours before battery adoption, and then after they added batteries they further reduce electricity demand during these hours.

There is no increase in electricity exported back to the grid during peak hours, which could be due to battery capacity constraints because all battery electricity is used to power the house at a given hour during the peak hours and there is no excess battery electricity to send back to the grid. E26 consumers’ electricity consumption patterns are consistent with economic predictions in the winter by reducing electricity delivered during the entire peak hours. However, in the summer, E26 consumers only reduce electricity delivered from 2 to 5 p.m. instead of during the entire peak hours from 2 to 8 p.m. Instead, E26 consumers reduce electricity delivered from 10 p.m. to 5 a.m. Such consumption patterns of E26 consumers in the summer are not fully consistent with economic predictions because they can shift some of the electricity reduction from 10 p.m.–5 a.m. to 5–8 p.m. so that they can save more on their electricity bills.

In this section, we show that on average, two groups of consumers—E26 and E21 consumers—exhibit some electricity consumption patterns that are not maximizing their economic benefits. We acknowledge that consumers self-select into different rate plans and that consumers on different rate plans will exhibit different behavior patterns. We use the consumer-fixed effects to control for any time-invariant unobserved factors that can cause the selection into price plans. In the next section, we examine heterogeneous individual consumer electricity consumption patterns.

### Heterogeneous electricity consumption patterns by individual consumers

**Figure 5:**
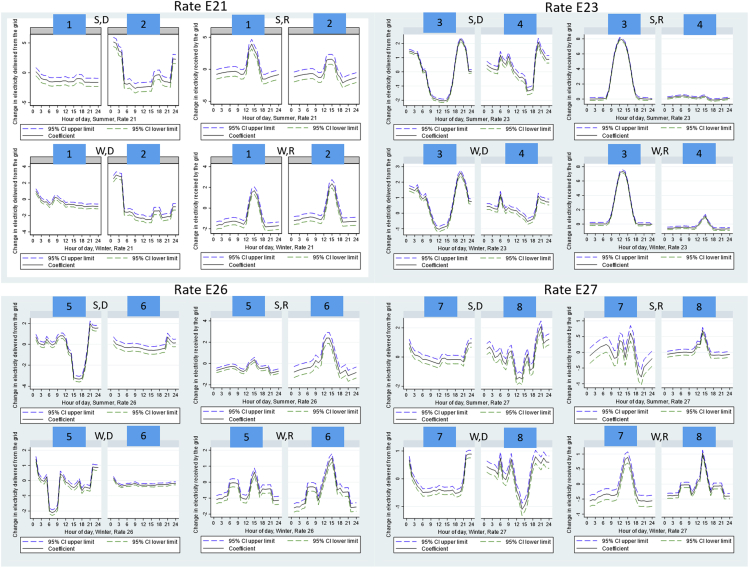
Impact of battery adoption on electricity delivered (purchases) or received (exports), eight individual consumers Each figure is a regression result of an individual consumer. The number in each blue box is the consumer ID number. y axis is the change in hourly electricity measured in kWh/hour due to battery adoption; x axis is the hour-of-day. The black solid line indicates point estimates; the blue and green dashed lines indicate 95% confidence intervals. “S” indicates summer months (May–Oct); “W” indicates winter months (Jan–Apr, Nov–Dec); “D” indicates electricity delivered from the grid to the consumer; “R” indicates electricity received by the grid from the consumer.

For each rate plan, we pick two consumers to illustrate the heterogeneity. We randomly picked these consumers instead of analyzing the electricity consumption patterns of each consumer of the whole sample because our main goal is to illustrate the differences in electricity consumption profiles instead of understanding each individual consumer’s profile. The results of this section are not intended to be generalizable to a broader consumer group. Figure 5 shows the results of running regressions of an individual battery consumer (instead of a group of consumers). These are the regressions for a single consumer, without any control consumers. Rate plan E21 has 3–6 p.m. as the peak hours all year round. Consumer 1 under rate plan E21 exhibits consumption patterns that cannot be fully predicted by economic or engineering models. This consumer reduces the electricity delivered not only during peak hours to avoid purchasing electricity at a higher cost but also during other off-peak hours. A consumer that maximizes bill savings can shift some reduction in electricity delivered from off-peak hours to peak hours to further reduce electricity bills. However, such behavior could be because the consumers want to consume solar electricity throughout the day instead of just during peak hours. In terms of electricity received, this consumer increases electricity exported back to the grid during the peak hours but also the 3 h before 3 p.m. This is only partially consistent with economic predictions because consumers can shift some increase in electricity received from the off-peak hours to the peak hours to increase solar electricity revenue.

Consumer 2 exhibits consumption patterns that are partially consistent with economic predictions. The consumer increases electricity delivered at night so that batteries can charge at night to utilize the lower prices. This behavior may increase environmental pollution because electricity at night is mostly powered by baseload fossil fuel power plants which can generate higher carbon and air emissions. The consumer does not have a statistically significant reduction in electricity delivered during the peak hours but reduces electricity during the hours before the peak hours from 6 a.m. to 3 p.m., which is not fully maximizing the economic benefits because the consumer can shift some of the reduction in these hours to 3–6 p.m. to further reduce electricity bills. In terms of electricity received, similar to consumer 1, this consumer increases electricity exported back to the grid during the peak hours but also the 3 h before 3 p.m. which is only partially maximizing the benefits.

Rate E23 is the non-TOU price plan. Consumer 3 exhibits rebound effects. In both winter and summer, consumer 3 reduces electricity delivered during the day and increases electricity delivered at night and in the evening hours. This implies that batteries are charging at night and then powering the house during the day. The increase in electricity delivered during early evening hours implies potential rebound effects of increasing electricity consumption during the evening.

Consumer 4 exhibits rebound effects. The consumer increases electricity delivered in the morning and at night and reduces electricity delivered in the late afternoon and early evening, which implies that the consumer uses battery electricity (charged from both grid electricity at night and solar electricity) to power the house in the late afternoon and early evening hours. The electricity received does not change much, implying rebound effects in total electricity consumption given on average, electricity delivered increases.

Both rate E26 and E27 are TOU price plans with 2–8 p.m. as peak hours in the summer, and 5–9 a.m. and 5–9 p.m. as peak hours in the winter. For E26 consumers, consumer 5 exhibits consumption patterns that are generally consistent with economic predictions in both winter and summer. The consumer reduces electricity delivered in the one peak hour window in the summer and in the two peak hour windows in the winter. Consumer 6 has almost no changes in electricity delivered at all hours in the summer.

For E27 consumers, consumer 7 exhibits consumption patterns partially consistent with economic predictions by increasing electricity delivered at night to charge the battery and increasing electricity received during some peak hours, but the hours can be re-optimized to better match the peak and off-peak hours. Consumer 8 also exhibits patterns partially consistent with economic predictions. The consumer reduces electricity delivered around noon and in the early afternoon and increases electricity delivered at night and in the early morning hours. This implies that the battery is charging at night and in the early morning hours with grid electricity, and the battery is discharging (charged with both grid and solar electricity) to power the house around noon and in the early afternoon hours. Such a pattern is only partially consistent with economic predictions because the hours when the electricity is delivered do not fully match the peak hours schedules in the winter and the summer.

## Discussion

Policymakers need to assess the social and private benefits of battery storage and solar PV to design the most efficient incentive programs for these technologies. Most current policy discussions are based on engineering, economics simulation, and techno-economics studies. As we demonstrate in this study, PV and battery co-adopters’ battery charging and discharging profiles and electricity consumption patterns are heterogeneous and can differ from the paths as predicted by standard economics and engineering theories. This necessitates accurate empirical impact evaluation. To the best of our knowledge, there have been no empirical studies examining the actual impact of adopting battery-storage technologies and our study fills this gap.

Our result provides signalization of a potential problem in setting policies relating to battery storage and demand management. Our results of the deviations between actual and predicted electricity consumption patterns highlight the importance for utilities and policymakers to further understand the underlying behavioral reasons for such deviations in order to design more effective and better-targeted incentives and interventions (such as electricity pricing and nudges) to influence individual consumer behaviors so that their behaviors can maximize the total societal benefits (better resilience and reduced carbon emissions) from these technologies. For example, after adopting battery storage, some PV consumers do not reduce electricity purchased from the grid during peak hours nor increase electricity exported back to the grid during these hours. Policymakers should be aware of such discrepancies in actual versus predicted electricity consumption patterns when designing the optimal electricity price signals. Our results also imply that it is necessary to provide an information program to homeowners to educate them on the optimal charging and discharging profiles, both according to economics and social benefits standards. For consumers, our results will be beneficial for them to better understand the private benefits of technology co-adoption and thus help incentivize the adoption. For consumers already co-adopting these technologies, our results will be helpful for them to re-optimize their consumption patterns to enhance their private benefits and to maintain better thermal comfort.

Our sample consists of 56 consumers that added battery storage after adopting solar PVs. This sample is small and is also self-selected. Thus caution needs to be made when generalizing our results to the broader consumer groups. Our paper is one of the earliest efforts on an empirical assessment of the consequences of PV and battery co-adoption. Our results can potentially transform how engineers, utilities, and policymakers assess the impact and prospect of increasing diffusion of PVs and battery storage. Our results highlight the critical importance of such an empirical understanding of co-adopters’ heterogeneous electricity consumption patterns and an adequate evaluation framework. Our research can help improve relevant simulation modeling by combining engineering with empirical analysis.

### Limitations of the study

Our work provides empirical evidence of the heterogeneous changes in electricity consumption patterns of residential solar consumers due to adding battery storage units. Nonetheless, two limitations are worth noting. First, our sample size of 56 consumers is rather small and this sample is highly self-selected. This limits the generalizability of our results to a broader population. Future studies are needed in other geographical locations with larger sample sizes, in order to provide more representative results for relevant decision-makers. Second, we do not observe the actual consumer behaviors such as their battery settings, thermostat settings, occupancy, and usage of household appliances. We can only analyze the changes in electricity consumption patterns and cannot examine the underlying behavioral reasons for such changes. Future studies are needed such as interviews, surveys, or studies that collect the appliance and battery usage information. Answers to the behavioral questions will help utilities and policymakers to adopt a more targeted approach to maximize the social benefits of battery storage.

## STAR★Methods

### Key resources table

**Table:** 

REAGENT or RESOURCE	SOURCE	IDENTIFIER
**Deposited data**		

Hourly electricity usage data in Arizona	SPR	From SPR utility company directly
Hourly meteorological data	the National Oceanic and Atmospheric Administration (NOAA) Local Climatological Database	https://data.nodc.noaa.gov/cgi-bin/iso?id=gov.noaa.ncdc:C00684
Electricity price in Arizona	SPR Rate Book 2019	From SPR utility company directly
Battery and solar PV adoption information	SRP battery and solar study program	From SPR utility company directly

**Software and algorithms**		

STATA 15	This study	https://www.stata.com/stata15/

### Resource availability

#### Lead contact

Further information and requests for resources and reagents should be directed to and will be fulfilled by the lead contact, Yueming (Lucy) Qiu (yqiu16@umd.edu).

#### Materials availability

This study did not generate new unique reagents.

### Method details

#### Program and data

Our data comes from a major utility company called Salt River Project (SRP) which serves the Phoenix metropolitan area in Arizona. SRP started to offer rebate incentives at $300/kWh up to $3600 beginning May 2018 for residential consumers to install lithium-ion battery storage systems. We obtained access to the smart meter data of consumers that have participated in this incentive program. An average battery system sizes 7kWh in our study sample. There are a total of 396 battery homes in our sample that adopted the batteries between 2018 and 2019, the majority (383) of which are solar homes with battery units. During our study period, battery storage units in SRP were allowed to discharge electricity to the grid. These battery consumers can program their storage units instantly using the smart control devices installed on the batteries or via the consumers’ user portals. Consumers can choose pre-programmed settings to have the batteries automatically charge and discharge at certain hours. Unfortunately, we do not have the data on the pre-programmed settings or the information on how many customers modified the pre-programmed settings. If consumers’ battery storage units are also managed by third-party demand-side management companies, then they will be less likely to modify the settings. Otherwise, consumers may likely change the pre-programmed settings. We do not have the information on how the battery energy management systems (EMS) work in our data sample. The observed discrepancy between the actual electricity consumption patterns and that predicted by simulations could also be partially explained by how EMS works, which is more of a deterministic approach (Fett et al., 2021). The implication of this lack of information is that we cannot precisely attribute the observed heterogeneity in electricity consumption patterns to particular determinants such as battery functionality, consumers’ own decisions on battery discharging and charging profiles, and the control of demand-side management companies (if any).

We remove the households that expanded the PV units (67 households) during our study time. We also exclude the few households that expanded their battery system after adopting the first battery unit (10 households). Also since we are interested in how adding batteries changes the behaviors of PV consumers, we only focus on the PV consumers that adopted the batteries after first installing PVs. In other words, we remove the consumers that installed PV and batteries at the same time (250 households). In our final analysis sample, there are 56 PV consumers (396-13-67-10-250) who added battery storage units at a later time and these consumers form our treatment group. SRP initially provided us with 27,560 non-battery PV consumers. We randomly selected 5% of these consumers for the purpose of reducing computational time, which gives us 1378 non-battery PV consumers as the control group. Summary statistics of the electricity using patterns are shown in Table S1 in Supplementary Information.

The possibility of these PV battery consumers also having electric vehicles is low in our sample. This is because according to a recent residential energy technology survey conducted by the SRP, there are only 7% of solar consumers that have plug-in electric vehicles. Thus the confounding impact of EVs in our study will be small. In addition, according to (Liang et al., 2020a), about 11% of solar consumers have energy efficient ACs in Arizona, which makes the confounding impact of energy efficiency also relatively small in our study.

We acknowledge that the sample size of 56 battery consumers is small. This has implications in terms of external validity and generalizing the results of our sample to the broader population. However, given that our paper provides empirical evidence of the behaviors of battery and solar panel co-adopters, our research can help pave the way for further research once more adoption of batteries happens.

For all homes, we have their hourly smart meter electricity data from May 2013-April 2020 as well as their electricity price plans for each day. The battery storage adoption happened between May 2018 and June 2019 in our dataset. Thus we have the smart meter data for individual homes for both the pre-treatment and post-treatment periods, enabling a difference-in-differences analysis. The pre-treatment period is after PV adoption and before battery adoption. The hourly smart meter data is resampled from the 15-min interval data, meaning that we take the average of the four recorded demands at each 15-min interval of a given hour, in order to reduce the computing time when running the regression analyses.

The smart meter data records both hourly electricity delivered to the consumer and hour electricity exported back to the grid (also called electricity received by the grid). All PV consumers are on the net-metering plan in our sample. Under a net-metering plan in our study region, when solar panels generate electricity, solar electricity is first being used to power the house. If there is any excess solar electricity left, PV consumers will export solar electricity back to the grid and they will receive credits against their bills for the electricity exported to the grid at the same retail marginal electricity price they will need to pay the utility at a given hour. After consumers adopt batteries, batteries can charge using either grid or solar electricity. Batteries can discharge to power the house or export electricity back to the grid.

The battery consumers are on several price plans with different on-peak and off-peak hours and price levels. Consumers self-selected into these plans. Since the hourly marginal electricity prices determine the opportunity cost of using electricity at a given hour, consumers on different price plans will exhibit different electricity using behaviors (Liang, 2020b; Qiu et al., 2018). We thus divide our analysis by rate plans. Out of the 56 battery consumers, five consumers are on a price plan called E21 time-of-use rate (TOU), 12 consumers are on E23 increasing block rate (non-TOU rate), 12 consumers are on E26 TOU rate, and 24 are on E27 TOU rate. These consumers stay at their specific rates during our study time period. The E27 plan has an on-peak demand (kW) charge (Carroll, 2018). Consumers on the rate plan with a demand charge could have different electricity consumption patterns. For example, they might pay more attention to their usage during peak hours. The remaining three consumers are on three other plans and we do not analyze these three consumers separately; we include these three consumers when we analyze the overall sample. Details of these price plans, with the hours of peak and off-peak periods and marginal prices for each hour, can be found in Table S2 in the supplementary information.

#### Summary of the empirical strategy

Our central research question is how battery storage adoption changes the electricity consumption patterns of residential solar PV consumers. The potential endogeneity issues can arise from the following factors which can bias our estimation of the impact of battery adoption. First, PV consumers who self-selected to adopt batteries might have characteristics that are different from non-battery PV consumers. For example, these battery consumers might have higher incomes, or they might be more technology-savvy. These characteristics can be hard to observe and can also influence their electricity using patterns. Second, there can be other contemporaneous changes that happen around the same time when the solar consumers added batteries, such as other technology changes or household changes. These contemporaneous changes also impact consumers’ electricity using behaviors and thus can bias our causal estimation of the impact of battery adoption. Earlier we discussed that the probability of solar consumers adopting batteries and EVs at the same time or adopting batteries and energy efficiency at the same time is low, which implies the likely low confounding influence from contemporaneous technology adoption. If the co-adoption of EVs with battery storage happens, this will cause our estimated rebound effects due to adding battery storage to be over-estimated since EVs can also cause an increase in electricity consumption.

We adopt the following strategies to deal with the above-mentioned issues. First, we use a difference-in-differences (DID) method and panel regressions to control for time-invariant consumer-level unobservable characteristics such as consumer socio-demographic attributes, environmental awareness, and technology attitudes that can influence both their decision to adopt battery units and the electricity using behaviors. All control groups have solar PVs but no batteries. Second, we conduct an event study analysis to confirm the parallel trend assumption to make sure the battery and non-battery consumers share similar trends of electricity using behaviors prior to the adoption of batteries. Third, in addition to the randomly selected non-battery solar consumers, we also use propensity score matching (PSM) (Dehejia and Wahba, 2002) and coarsened exact matching (CEM) (Iacus et al., 2012) on pre-treatment electricity using patterns to create another control group that shares similar levels of electricity consumption with the battery consumers in the pre-treatment period. Fourth, to eliminate other technology changes such as expanding solar panel sizes, we remove the consumers that have expanded PV sizes from our sample. Lastly, we conduct two robustness checks. We add a group-specific time trend (an interaction term between the treatment group indicator and the day-of-sample variable) to control for any potential differential trends in the pre-treatment period, a method similar to the one used in Davis et al. (2014). The time trend can also help control for any time-variant unobservable differences between the battery and non-battery consumers, such as contemporaneous changes that can happen around the same time as adopting batteries. The second robustness check is to only use the battery consumers without any control consumers in the analysis, to eliminate any remaining concerns about the systemic differences between the battery and non-battery consumers. The results of the robustness checks are consistent with our main results (see Figure S1 and Table S7).

#### The base panel regression model

We use a two-way fixed effects panel regression model for the DID analysis. In our analysis, all consumers have adopted solar panels. The pre-treatment period is after PV adoption and before battery adoption. The post-treatment period is after PV consumers adopt batteries. A base model is as follows:

(Equation 1) $${\text{kWh}}_{\text{ih}}={\text{α}}_{\text{i}}+\sum_{\text{H}=1}^{24}{\text{β}}_{\text{H}}{\text{Battery}}_{\text{ih}}\text{∗}{\text{I}}_{\text{H}}+\text{γ}{\text{p}}_{\text{ih}}+\text{f}\left({\text{HDD}}_{\text{ih}}\right)\text{θ}+\text{f}\left({\text{CDD}}_{\text{ih}}\right)\text{η}+\text{ Hour}\;\text{of}\;\text{day}+\text{day}\;\text{of}\;\text{sample}+{\text{ε}}_{\text{ih}}$$

where kWh measures the hourly electricity delivered to the PV consumer or the hourly electricity exported to the grid by the PV consumer; we examine the hourly electricity delivered (purchases) and received (exports) in two separate analyses. We are not estimating a regression for the subsample of household-hours that are net consumers, nor are we estimating the same regression for the subsample of household-hours that are net producers. Instead, all household-hours appear in both regressions. In a given hour, the electricity delivered and received can be both positive numbers since we are aggregating the 15-min smart meter data within an hour. In an hour when a consumer does not purchase any electricity from the grid, the electricity delivered variable will be zero. Similarly, in an hour when a consumer does not send any electricity back to the grid, the electricity received variable will be zero. As a result, we do not have this type of sample selection problem. We are not taking the log of kWh because we are interested in the absolute magnitudes of the changes in electric load, instead of percentage changes, similar to the approach taken in existing studies such as (Burkhardt et al., 2019; Qiu et al., 2022).

The subscript _i_ indicates individual customer; _h_ indicates hour of sample; H indicates hour of day. I_H_ is an indicator variable for each hour of day. Battery_ih_ is equal to one if customer i has a battery installed at h. p_ih_ is the marginal electricity price for customer i at h. HDD is heating degree days as calculated by 65 - temperature; CDD is cooling degree days as calculated by temperature - 65; f is spline function for HDD and CDD. Hour-of-day fixed effects control for hourly differences in electricity using patterns within a day. Our rich dataset allows us to use day-of-sample (year-by-day-of-year) fixed effects, which can control for variations in electricity consumption patterns on each day of our sample including daily and seasonal patterns that vary across years. Note that we do not include weekend or holiday indicators because these two indicators will be automatically dropped with the day-of-sample fixed effects included, which already capture the effects of a weekend or a holiday. The standard errors in all our models are clustered at the individual household level.

We do not normalize the electricity consumption curves in our analysis because DID eliminates any potential bias that can be generated from differences in the levels of consumption. In other words, the DID approach is comparing the changes in the hourly electricity demand of a given consumer before and after the adoption of battery storage. This will difference out any pre-existing differences in the levels of consumption at any given hour across different consumers. DID is not comparing the load curves across different consumers.

#### Matching

In order to further eliminate any potential systematic differences between the battery and the non-battery consumers, we use PSM and CEM to find control groups that are similar in terms of electricity using patterns in the pre-treatment period. We first randomly assign a hypothetical battery adoption date for each control consumer. Then, for each treatment and control consumer, we calculate the average pre-treatment summer and winter hourly electricity delivered and received. Based on these four variables, we match each treatment consumer with similar control consumers. We run PSM and CEM separately. The PSM first regresses the battery adoption status on the four matching variables and obtains the predicted probability of battery adoption. In the second stage, PSM matches the treatment and control consumers based on the distance in the predicted probability of adopting a battery. We use matching with caliper set at 0.25 SD. The CEM process first divides each matching variable into several strata and then conducts exact matching based on each stratum. We use Stata’s default binning algorithm to automatically coarsen the data, which gives 12 cutoff points for each variable when dividing data into strata. The PSM process leaves 56 battery consumers and 972 control consumers. The CEM process leaves 37 battery consumers and 199 control consumers.

Table S3 in the supplementary information shows the balancing results and Table S3 confirms that the matched control and treatment groups are comparable. Table S3 also shows that the matched sample performs better compared to the unmatched sample in terms of the sample mean difference and the variance distribution. We then run regression models using the matched control and treatment groups from PSM and CEM. We acknowledge that matching is only based on observable characteristics and there can be unobserved differences between households with and without batteries that cannot be controlled for by the matching strategy. Thus we combine matching with DID to eliminate time-invariant unobserved factors that can bias the results.

#### Event study

We conduct an event study analysis to check for the parallel trend assumption (the battery consumers and non-battery consumers are comparable in terms of exhibiting similar trends of electricity using patterns prior to the treatment) and to examine the average change in hourly electricity delivered and received after adopting batteries. The following event study model is applied:

(Equation 2) $${\text{kWh}}_{\text{ih}}={\text{α}}_{\text{i}}+\sum_{\text{w}=-\text{n}}^{\text{n}}{\text{β}}_{\text{W}}1{\left[\text{Time}\;\text{to}\;\text{battery}\;\text{adoption}=\text{w}\right]}_{\text{ih}}+\text{γ}{\text{p}}_{\text{ih}}+\text{f}\left({\text{HDD}}_{\text{ih}}\right)\text{θ}+\text{f}\left({\text{CDD}}_{\text{ih}}\right)\text{η}+\text{Day}\;\text{of}\;\text{sample}+\text{ Hour}\;\text{of}\;\text{day}+{\text{ε}}_{\text{ih}}$$

where h indicates the hour of sample; 1[Time to battery adoption = ]_ih_ is a dummy variable indicating the week to the battery adoption which the hour-of-sample d falls into. For example, if the dummy variable is indicating 5 days prior to adopting the battery, then this variable is equal to one if it is 5 days prior to the adoption and is equal to 0 if it is another day. Other variables share the same definitions as in Equation (1). Following (Burlig et al., 2021), in the event study analysis, we look at the average hourly electricity consumption instead of the consumption by hour of day.

## Data Availability

• Data: Climate factors are obtained from U.S. Local Climatological Data (LCD) at https://data.nodc.noaa.gov/cgi-bin/iso?id=gov.noaa.ncdc:C00684. The high-frequency electricity data in Arizona are from the SRP. For SRP’s data, as restricted by a non-disclosure agreement, they are available from the authors upon reasonable request and with permission from the SRP. All data and models are processed in Stata 15.0. • Code: All custom code is available on GitHub from https://github.com/LucyEnergy/Battery_PV. • Any additional information required to reanalyze the data reported in this paper is available from the lead contact upon request.
