# Supplementary material for: Heterogeneous changes in electricity consumption patterns of residential distributed solar consumers due to battery storage adoption
Source: iScience. 2022 May 4;25(6):104352. doi: 10.1016/j.isci.2022.104352 (PMC9121249; doi:10.1016/j.isci.2022.104352)
Supplement: Document S1. Figure S1 and Tables S1–S7 [file mmc1.pdf]

iScience, Volume *25*

Supplemental information

Heterogeneous changes in electricity consumption patterns of residential distributed solar consumers due to battery storage adoption

Yueming (Lucy) Qiu, Bo Xing, Anand Patwardhan, Nathan Hultman, and Huiming Zhang

Supplementary Information

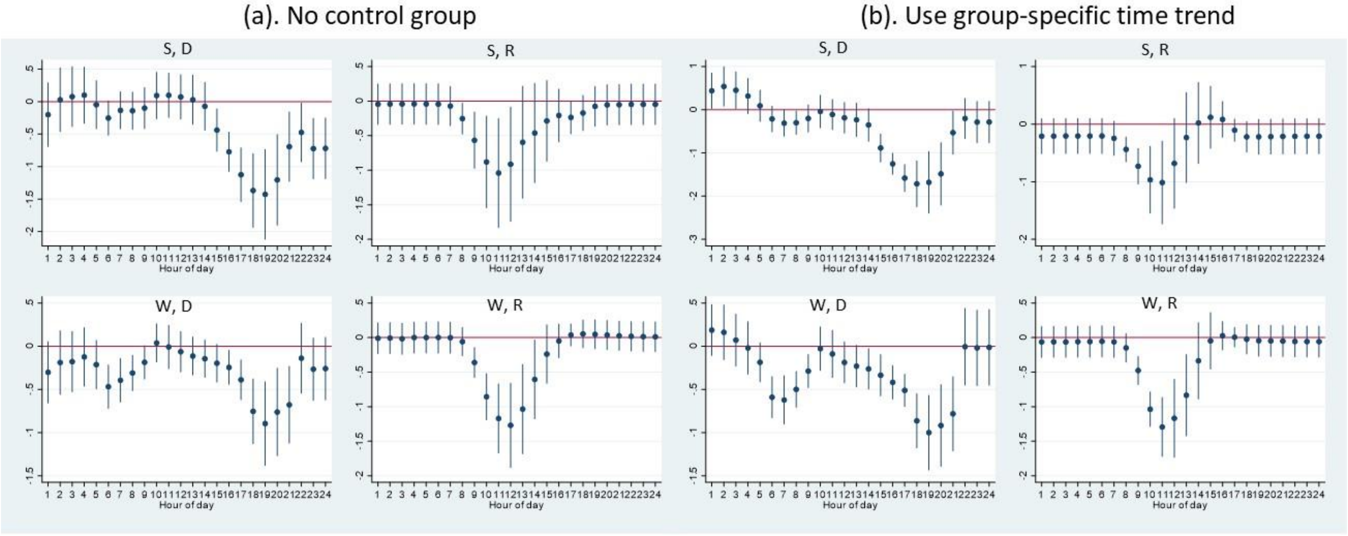

Figure S1. Impact of battery adoption on electricity delivered (purchases) or received (exports), robustness checks. Related to STAR Methods.

Notes: Panel (a) only uses the battery and PV co-adopters in the regressions and no PV-only consumers. Panel (b) includes group-specific time trends in the regressions. y-axis is the change in hourly electricity measured in kWh/hour due to battery adoption; x-axis is the hour-of-day. The dots indicate point estimates and the vertical lines indicate 95% confidence intervals. "S" indicates summer months (May-Oct); "W" indicates winter months (Jan-Apr, Nov-Dec); "D" indicates electricity delivered from the grid to the consumers; "R" indicates electricity received by the grid from the consumers. Detailed coefficients can be found in Table S7 in Supplementary Information.

Table S1. Summary statistics. Related to STAR Methods.

Randomly selected control consumers: non-battery PV consumers						
Variable		Obs	Mean	Std. Dev.	Min	Max
Summer	Electricity delivered	17,775,597<br>n=1378, t=12,900 [a]	1.95	1.95	0.00	47.20
	Electricity received	8,461,255<br>n=1378,t=6,140	0.63	1.31	0.00	19.92
Winter	Electricity delivered	19,341,633<br>n=1378,t=14,036	1.13	1.41	0.00	157.96
	Electricity received	11,107,019<br>n=1378,t=8,060	0.70	1.48	0.00	21.10
Treatment consumers: battery and PV consumers						
Variable		Obs	Mean	Std. Dev.	Min	Max
Pre-battery-adoption period						
Summer	Electricity delivered	795,719<br>n=56, t=14,209	2.13	2.33	0.00	31.51
	Electricity received	421,743<br>n=56, t=7,531	0.78	1.50	0.00	11.60
Winter	Electricity delivered	780,812<br>n=56, t=13,943	1.27	1.60	0.00	22.93
	Electricity received	450,926<br>n=56, t=8,052	0.75	1.52	0.00	11.95
Post-battery-adoption period						
Summer	Electricity delivered	163,645<br>n=56, t=2,922	1.88	2.38	0.00	25.03
	Electricity received	147,901<br>n=56, t=2,641	0.57	1.61	0.00	13.11
Winter	Electricity delivered	256,108<br>n=56, t=4,573	1.20	1.83	0.00	25.04
	Electricity received	243,053<br>n=56, t=4,340	0.51	1.54	0.00	12.97

Notes: [a] *n* means the number of consumers; *t* means the number of hours on average per consumer available in the dataset

Table S2. Details of marginal prices for each rate plan. Related to STAR Methods.

Pricing plan	Name	Division	Summer months (May, Jun, Sep, Oct)	Summer peak months (Jul, Aug)	Winter months (other months)	Notes
E21	Super peak time-of-use service	On-peak	$0.2895	$0.3444	$0.1063	On-peak hours year-round consist of those hours from 3 p.m. to 6 p.m., Monday through Friday. All other hours are off-peak
		Off-peak	$0.0829	$0.0853	$0.0738	
E23	Standard price plan for residential service (non-TOU)	0-2,000 kWh	$0.1091	$0.1157	$0.0782	Increasing block rate; no intra-day variation in marginal electricity price
		2,001+ kWh	$0.1134	$0.1270		
E26	Standard price plan for residential time-of-use service	On-peak	$0.2094	$0.2409	$0.0951	Summer on-peak hours from 2 p.m. to 8 p.m., Monday through Friday; winter on-peak hours from 5 a.m. to 9 a.m. and from 5 p.m. to 9 p.m., Monday through Friday, all other hours are off-peak.
		Off-peak	$0.0727	$0.0730	$0.0691	
E27	Generation price plan	On-peak	$0.0462	$0.0622	$0.0410	Summer on-peak hours from 2 p.m. to 8 p.m., Monday through Friday; winter on-peak hours from 5 a.m. to 9 a.m. and from 5 p.m. to 9 p.m., Monday through Friday; super off-peak hours are from 11 p.m. to 5 a.m. All hours that are not on-peak or super off-peak are off-peak.
		Off-peak	$0.0360	$0.0412	$0.0370	

Table S3. Balance checks for PSM (We do not show the balance checks for CEM because by construction, CEM conducts exact matching on strata). Related to STAR Methods.

	Unmatched /matched	Mean Treated	Control	% reduction %bias	|bias|	t-test t	p>|t|	V(T) /V(C)
Winter, Delivered	U	1.3446	1.1388	28.5		2.08	0.038	1.01
	M	1.3446	1.3362	1.2	95.9	0.05	0.958	0.61
Summer, Delivered	U	2.274	1.9631	28		2.42	0.016	1.97*
	M	2.274	2.167	9.6	65.6	0.44	0.658	1.02
Winter, Received	U	0.73166	0.69092	9.3		0.66	0.51	0.87
	M	0.73166	0.66077	16.2	-74	0.86	0.394	0.86
Summer, Received	U	0.70057	0.62689	16.4		1.2	0.231	1.02
	M	0.70057	0.69304	1.7	89.8	0.09	0.932	0.87

if variance ratio outside [0.85; 1.18] for U and [0.85; 1.18] for M

Table S4(a). Regression results for Figure 2, panel (a) no matching. Related to Figure 2.

	Summer, Delivered	Summer, Received	Winter, Delivered	Winter, Received
Battery* Hour 1	0.519***	0.090***	0.184	-0.721***
	(0.186)	(0.008)	(0.139)	(0.017)
Battery* Hour 2	0.619***	0.103***	0.171	-0.720***
	(0.204)	(0.009)	(0.139)	(0.016)
Battery* Hour 3	0.539***	0.104***	0.090	-0.722***
	(0.193)	(0.008)	(0.129)	(0.016)
Battery* Hour 4	0.407**	0.109***	0.010	-0.722***
	(0.184)	(0.009)	(0.130)	(0.015)
Battery* Hour 5	0.206	0.115***	-0.145	-0.723***
	(0.160)	(0.009)	(0.088)	(0.015)
Battery* Hour 6	-0.104	0.128***	-0.490***	-0.723***
	(0.124)	(0.008)	(0.072)	(0.016)
Battery* Hour 7	-0.178	0.091***	-0.510***	-0.723***
	(0.114)	(0.013)	(0.088)	(0.016)
Battery* Hour 8	-0.149	-0.098***	-0.405***	-0.659***
	(0.123)	(0.034)	(0.088)	(0.076)
Battery* Hour 9	-0.089	1.421***	-0.224**	-0.381
	(0.155)	(0.065)	(0.099)	(0.317)
Battery* Hour 10	0.069	3.750***	-0.017	0.390
	(0.195)	(0.090)	(0.135)	(0.819)
Battery* Hour 11	0.005	5.192***	-0.073	1.545
	(0.184)	(0.108)	(0.151)	(1.003)
Battery* Hour 12	-0.069	5.351***	-0.156	3.595***
	(0.186)	(0.120)	(0.145)	(0.071)
Battery* Hour 13	-0.126	4.885***	-0.211	4.148***
	(0.195)	(0.126)	(0.143)	(0.535)
Battery* Hour 14	-0.260	4.989***	-0.248*	3.867***

	(0.196)	(0.118)	(0.137)	(0.323)
Battery* Hour 15	-0.705***	4.273***	-0.315**	3.138***
	(0.165)	(0.096)	(0.134)	(0.322)
Battery* Hour 16	-1.052***	1.686***	-0.397***	1.329***
	(0.115)	(0.068)	(0.103)	(0.511)
Battery* Hour 17	-1.319***	0.186***	-0.481***	0.263
	(0.120)	(0.039)	(0.077)	(0.426)
Battery* Hour 18	-1.408***	-0.180***	-0.771***	-0.472**
	(0.215)	(0.018)	(0.122)	(0.226)
Battery* Hour 19	-1.378***	-0.081***	-0.888***	-0.715***
	(0.298)	(0.007)	(0.176)	(0.014)
Battery* Hour 20	-1.157***	-0.032***	-0.777***	-0.723***
	(0.297)	(0.003)	(0.195)	(0.018)
Battery* Hour 21	-0.415*	0.000	-0.659***	-0.723***
	(0.220)	(.)	(0.179)	(0.019)
Battery* Hour 22	-0.134	0.033***	-0.064	-0.723***
	(0.201)	(0.003)	(0.188)	(0.019)
Battery* Hour 23	-0.208	0.056***	-0.070	-0.722***
	(0.233)	(0.005)	(0.192)	(0.018)
Battery* Hour 24	-0.206	0.073***	-0.065	-0.721***
	(0.233)	(0.006)	(0.192)	(0.017)
Electricity price	-1.578***	3.623***	-1.249*	-3.389
	(0.237)	(0.113)	(0.736)	(5.151)
Constant	1.421***	-0.125*	1.372***	0.186
	(0.041)	(0.074)	(0.062)	(0.386)
HDD control	Yes	Yes	Yes	Yes
CDD control	Yes	Yes	Yes	Yes
Fixed effects				
Household	Yes	Yes	Yes	Yes
Day of sample	Yes	Yes	Yes	Yes
Hour of day	Yes	Yes	Yes	Yes

N	18734961	1193552	20378553	3255133
Standard errors in parentheses	*p<0.1	**p<0.05	***p<0.01	

Table S4(b). Regression results for Figure 2, panel (b) no matching, weekdays only. Related to Figure 2.

	Summer, Delivered	Summer, Received	Winter, Delivered	Winter, Received
Battery* Hour 1	0.546***<br>(0.190)	0.283***<br>(0.021)	0.209<br>(0.140)	-0.682***<br>(0.014)
Battery* Hour 2	0.647***<br>(0.213)	0.297***<br>(0.021)	0.183<br>(0.144)	-0.683***<br>(0.014)
Battery* Hour 3	0.558***<br>(0.194)	0.299***<br>(0.022)	0.093<br>(0.133)	-0.684***<br>(0.015)
Battery* Hour 4	0.423**<br>(0.179)	0.302***<br>(0.022)	0.003<br>(0.129)	-0.684***<br>(0.015)
Battery* Hour 5	0.198<br>(0.159)	0.306***<br>(0.022)	-0.164*<br>(0.088)	-0.685***<br>(0.015)
Battery* Hour 6	-0.106<br>(0.129)	0.320***<br>(0.022)	-0.568***<br>(0.094)	-0.685***<br>(0.015)
Battery* Hour 7	-0.206*<br>(0.122)	0.286***<br>(0.022)	-0.600***<br>(0.117)	-0.684***<br>(0.014)
Battery* Hour 8	-0.194<br>(0.130)	0.108***<br>(0.036)	-0.477***<br>(0.093)	-0.623***<br>(0.059)
Battery* Hour 9	-0.094<br>(0.169)	1.546***<br>(0.062)	-0.265**<br>(0.103)	-0.320<br>(0.317)
Battery* Hour 10	0.066<br>(0.209)	4.054***<br>(0.086)	-0.006<br>(0.148)	0.494<br>(0.831)
Battery* Hour 11	-0.004<br>(0.194)	5.324***<br>(0.101)	-0.067<br>(0.159)	1.623<br>(1.074)
Battery* Hour 12	-0.080<br>(0.199)	5.591***<br>(0.114)	-0.165<br>(0.145)	3.610***<br>(0.142)
Battery* Hour 13	-0.127<br>(0.208)	4.687***<br>(0.122)	-0.208<br>(0.146)	4.195***<br>(0.574)

Battery* Hour 14	-0.246	4.951***	-0.240*	3.870***
	(0.202)	(0.110)	(0.140)	(0.315)
Battery* Hour 15	-0.779***	4.292***	-0.313**	3.180***
	(0.170)	(0.086)	(0.138)	(0.324)
Battery* Hour 16	-1.146***	1.426***	-0.396***	1.233**
	(0.115)	(0.057)	(0.104)	(0.479)
Battery* Hour 17	-1.475***	0.331***	-0.489***	0.290
	(0.140)	(0.025)	(0.076)	(0.448)
Battery* Hour 18	-1.609***	0.000	-0.841***	-0.449*
	(0.250)	(.)	(0.128)	(0.240)
Battery* Hour 19	-1.574***	0.105***	-0.978***	-0.675***
	(0.342)	(0.017)	(0.188)	(0.022)
Battery* Hour 20	-1.379***	0.158***	-0.895***	-0.684***
	(0.346)	(0.020)	(0.208)	(0.014)
Battery* Hour 21	-0.425*	0.191***	-0.760***	-0.685***
	(0.232)	(0.020)	(0.189)	(0.014)
Battery* Hour 22	-0.096	0.224***	0.017	-0.684***
	(0.219)	(0.020)	(0.211)	(0.014)
Battery* Hour 23	-0.180	0.249***	0.003	-0.683***
	(0.248)	(0.020)	(0.218)	(0.014)
Battery* Hour 24	-0.179	0.266***	0.009	-0.682***
	(0.248)	(0.021)	(0.218)	(0.014)
Electricity price	-1.409***	4.016***	-1.677**	-1.456
	(0.250)	(0.126)	(0.732)	(5.086)
Constant	1.410***	-0.159**	1.401***	-0.035
	(0.042)	(0.075)	(0.062)	(0.384)
HDD control	Yes	Yes	Yes	Yes
CDD control	Yes	Yes	Yes	Yes
Fixed effects				
Household	Yes	Yes	Yes	Yes
Day of sample	Yes	Yes	Yes	Yes

Hour of day	Yes	Yes	Yes	Yes
N	13411814	852114	14539741	2315743

Standard errors in parentheses *p<0.1 **p<0.05 ***p<0.01

Table S4(c). Regression results for Figure 2, panel (c) PSM. Related to Figure 2.

	Summer, Delivered	Summer, Received	Winter, Delivered	Winter, Received
Battery* Hour 1	0.060	0.023	-0.041	0.007
	(0.227)	(0.094)	(0.151)	(0.060)
Battery* Hour 2	0.025	0.025	-0.097	0.009
	(0.186)	(0.094)	(0.134)	(0.060)
Battery* Hour 3	-0.015	0.026	-0.124	0.003
	(0.160)	(0.094)	(0.124)	(0.061)
Battery* Hour 4	-0.031	0.027	-0.145	0.016
	(0.141)	(0.094)	(0.111)	(0.060)
Battery* Hour 5	-0.152	0.026	-0.245**	0.016
	(0.125)	(0.094)	(0.097)	(0.060)
Battery* Hour 6	-0.241*	0.025	-0.521***	0.020
	(0.134)	(0.094)	(0.085)	(0.060)
Battery* Hour 7	-0.212	-0.021	-0.556***	0.012
	(0.146)	(0.089)	(0.110)	(0.060)
Battery* Hour 8	0.015	-0.305***	-0.355***	-0.086
	(0.130)	(0.092)	(0.098)	(0.061)
Battery* Hour 9	0.206	-0.751***	-0.079	-0.468***
	(0.129)	(0.160)	(0.099)	(0.104)
Battery* Hour 10	0.450***	-1.153***	0.229*	-1.075***
	(0.148)	(0.250)	(0.118)	(0.149)
Battery* Hour 11	0.421***	-1.405***	0.211*	-1.443***
	(0.150)	(0.292)	(0.125)	(0.205)
Battery* Hour 12	0.383**	-1.340***	0.148	-1.569***
	(0.161)	(0.291)	(0.119)	(0.234)
Battery* Hour 13	0.333*	-0.966***	0.077	-1.316***
	(0.172)	(0.294)	(0.129)	(0.230)
Battery* Hour 14	0.196	-0.686**	0.035	-0.846***

	(0.175)	(0.270)	(0.126)	(0.201)
Battery* Hour 15	-0.276	-0.439**	-0.040	-0.405**
	(0.183)	(0.221)	(0.128)	(0.168)
Battery* Hour 16	-0.664***	-0.250	-0.178	-0.123
	(0.150)	(0.153)	(0.119)	(0.111)
Battery* Hour 17	-1.111***	-0.153*	-0.421***	0.017
	(0.177)	(0.089)	(0.105)	(0.051)
Battery* Hour 18	-1.506***	-0.089	-0.915***	0.048
	(0.206)	(0.076)	(0.114)	(0.051)
Battery* Hour 19	-1.750***	-0.004	-1.172***	0.040
	(0.220)	(0.090)	(0.131)	(0.057)
Battery* Hour 20	-1.586***	0.014	-1.104***	0.033
	(0.199)	(0.093)	(0.143)	(0.058)
Battery* Hour 21	-0.757***	0.018	-0.960***	0.026
	(0.251)	(0.094)	(0.174)	(0.058)
Battery* Hour 22	-0.376	0.021	-0.191	0.019
	(0.266)	(0.094)	(0.226)	(0.058)
Battery* Hour 23	-0.362	0.021	-0.145	0.016
	(0.235)	(0.094)	(0.211)	(0.058)
Battery* Hour 24	-0.358	0.022	-0.138	0.015
	(0.235)	(0.094)	(0.211)	(0.059)
Electricity price	-1.133*	0.319	-1.371	-0.062
	(0.593)	(0.236)	(1.705)	(1.100)
Constant	1.927***	0.245***	1.844***	-0.007
	(0.174)	(0.061)	(0.209)	(0.105)
HDD control	Yes	Yes	Yes	Yes
CDD control	Yes	Yes	Yes	Yes
Fixed effects				
Household	Yes	Yes	Yes	Yes
Day of sample	Yes	Yes	Yes	Yes
Hour of day	Yes	Yes	Yes	Yes

N	1780656	967795	1916736	1197847

Standard errors in parentheses *p<0.1 **p<0.05 ***p<0.01

Table S4(d). Regression results for Figure 2, panel (d) CEM. Related to Figure 2.

	Summer, Delivered	Summer, Received	Winter, Delivered	Winter, Received
Battery* Hour 1	0.361*	-0.033	0.063	-0.001
	(0.206)	(0.101)	(0.147)	(0.070)
Battery* Hour 2	0.484**	-0.031	0.047	0.000
	(0.243)	(0.101)	(0.142)	(0.070)
Battery* Hour 3	0.373*	-0.029	-0.034	-0.005
	(0.209)	(0.101)	(0.124)	(0.070)
Battery* Hour 4	0.184	-0.029	-0.143	0.005
	(0.142)	(0.101)	(0.092)	(0.070)
Battery* Hour 5	0.049	-0.029	-0.172**	0.005
	(0.125)	(0.101)	(0.083)	(0.070)
Battery* Hour 6	-0.148	-0.031	-0.490***	0.003
	(0.133)	(0.100)	(0.076)	(0.069)
Battery* Hour 7	-0.206	-0.086	-0.569***	-0.005
	(0.141)	(0.096)	(0.107)	(0.069)
Battery* Hour 8	0.007	-0.346***	-0.436***	-0.100
	(0.123)	(0.083)	(0.107)	(0.065)
Battery* Hour 9	0.183	-0.756***	-0.147	-0.452***
	(0.132)	(0.139)	(0.108)	(0.100)
Battery* Hour 10	0.434***	-1.134***	0.159	-1.064***
	(0.154)	(0.238)	(0.107)	(0.138)
Battery* Hour 11	0.375**	-1.339***	0.106	-1.438***
	(0.150)	(0.292)	(0.087)	(0.208)
Battery* Hour 12	0.316**	-1.170***	0.061	-1.497***
	(0.158)	(0.302)	(0.079)	(0.252)
Battery* Hour 13	0.276*	-0.768**	0.096	-1.275***
	(0.166)	(0.319)	(0.077)	(0.254)
Battery* Hour 14	0.165	-0.439	0.043	-0.780***

	(0.171)	(0.297)	(0.077)	(0.234)
Battery* Hour 15	-0.317*	-0.206	-0.028	-0.345*
	(0.168)	(0.234)	(0.085)	(0.193)
Battery* Hour 16	-0.655***	-0.109	-0.137*	-0.112
	(0.140)	(0.153)	(0.082)	(0.127)
Battery* Hour 17	-1.036***	-0.106	-0.320***	-0.006
	(0.164)	(0.076)	(0.082)	(0.057)
Battery* Hour 18	-1.393***	-0.087	-0.739***	0.019
	(0.210)	(0.071)	(0.096)	(0.061)
Battery* Hour 19	-1.642***	-0.047	-1.014***	0.014
	(0.227)	(0.097)	(0.117)	(0.069)
Battery* Hour 20	-1.476***	-0.045	-0.983***	0.009
	(0.189)	(0.100)	(0.127)	(0.069)
Battery* Hour 21	-0.638***	-0.037	-0.862***	0.005
	(0.217)	(0.101)	(0.139)	(0.069)
Battery* Hour 22	-0.217	-0.035	-0.116	0.002
	(0.215)	(0.101)	(0.221)	(0.069)
Battery* Hour 23	-0.122	-0.035	-0.103	-0.001
	(0.204)	(0.101)	(0.197)	(0.069)
Battery* Hour 24	-0.121	-0.034	-0.094	-0.001
	(0.204)	(0.101)	(0.198)	(0.070)
Electricity price	-2.333***	0.736**	-0.903	0.863
	(0.556)	(0.336)	(1.335)	(0.800)
Constant	1.628***	0.242***	1.420***	-0.071
	(0.072)	(0.062)	(0.112)	(0.086)
HDD control	Yes	Yes	Yes	Yes
CDD control	Yes	Yes	Yes	Yes
Fixed effects				
Household	Yes	Yes	Yes	Yes
Day of sample	Yes	Yes	Yes	Yes
Hour of day	Yes	Yes	Yes	Yes

N	8997548	4576784	9637888	5778289

Standard errors in parentheses *p<0.1 **p<0.05 ***p<0.01

Table S5 (a). Regression results for Figure 3, by rate, Rate E21 and Rate E23, No matching. Related to Figure 3.

	Rate E21				Rate E23			
	Summer, Delivered	Summer, Received	Winter, Delivered	Winter, Received	Summer, Delivered	Summer, Received	Winter, Delivered	Winter, Received
Battery* Hour 1	0.029	-0.058	0.288	0.085	0.413	-0.779**	-0.089	-0.367*
	(0.545)	(0.313)	(0.399)	(0.196)	(0.296)	(0.343)	(0.275)	(0.191)
Battery* Hour 2	-0.061	-0.055	0.307	0.086	0.656**	-0.779**	-0.128	-0.366*
	(0.582)	(0.312)	(0.462)	(0.196)	(0.265)	(0.343)	(0.154)	(0.191)
Battery* Hour 3	-0.168	-0.050	0.266	0.083	0.743**	-0.778**	-0.179	-0.368*
	(0.515)	(0.309)	(0.458)	(0.197)	(0.290)	(0.344)	(0.122)	(0.190)
Battery* Hour 4	-0.230	-0.050	0.254	0.091	0.882***	-0.778**	-0.031	-0.364*
	(0.506)	(0.308)	(0.456)	(0.195)	(0.313)	(0.344)	(0.158)	(0.192)
Battery* Hour 5	-0.622*	-0.050	-0.432**	0.092	0.813**	-0.778**	-0.140	-0.363*
	(0.323)	(0.308)	(0.213)	(0.195)	(0.396)	(0.344)	(0.126)	(0.192)
Battery* Hour 6	-0.533	-0.051	-0.554**	0.091	0.418	-0.779**	-0.337***	-0.363*
	(0.532)	(0.308)	(0.252)	(0.195)	(0.306)	(0.344)	(0.119)	(0.192)
Battery* Hour 7	-1.087***	-0.123	-0.521	0.081	0.086	-0.825**	-0.319	-0.371*
	(0.394)	(0.306)	(0.404)	(0.195)	(0.248)	(0.332)	(0.202)	(0.192)
Battery* Hour 8	-0.851***	-0.476	-0.373	-0.049	-0.111	-0.872***	-0.298	-0.443***
	(0.189)	(0.310)	(0.288)	(0.194)	(0.287)	(0.220)	(0.204)	(0.165)
Battery* Hour 9	-0.476***	-0.952***	-0.151	-0.464**	-0.106	-0.747	-0.250	-0.651***
	(0.132)	(0.292)	(0.338)	(0.222)	(0.323)	(0.482)	(0.274)	(0.168)
Battery* Hour 10	0.462	-1.114*	0.061	-1.021***	-0.083	-0.532	-0.333	-0.858**
	(0.451)	(0.610)	(0.416)	(0.375)	(0.304)	(0.860)	(0.217)	(0.402)

Battery* Hour 11	0.395	-1.337*	0.046	-1.540***	-0.119	-0.086	-0.451**	-0.827
	(0.397)	(0.763)	(0.320)	(0.566)	(0.290)	(0.965)	(0.210)	(0.656)
Battery* Hour 12	0.428	-1.323*	-0.074	-1.664**	-0.157	0.681	-0.489**	-0.287
	(0.436)	(0.788)	(0.309)	(0.669)	(0.299)	(0.950)	(0.221)	(0.785)
Battery* Hour 13	0.528	-1.008	-0.047	-1.274**	-0.297	1.112	-0.499**	0.267
	(0.541)	(0.722)	(0.327)	(0.643)	(0.278)	(0.942)	(0.207)	(0.778)
Battery* Hour 14	0.479	-0.795	-0.022	-0.667	-0.420	1.204	-0.526***	0.774
	(0.610)	(0.570)	(0.356)	(0.643)	(0.290)	(0.829)	(0.201)	(0.704)
Battery* Hour 15	0.414	-0.948***	0.037	-0.258	-0.776***	1.002	-0.569***	0.760
	(0.401)	(0.200)	(0.357)	(0.583)	(0.220)	(0.650)	(0.191)	(0.508)
Battery* Hour 16	-0.703***	-0.744***	-0.240	-0.105	-1.235***	0.356	-0.674***	0.346
	(0.107)	(0.172)	(0.307)	(0.361)	(0.219)	(0.407)	(0.164)	(0.276)
Battery* Hour 17	-0.802***	-0.500**	-0.412	0.037	-1.486***	-0.448**	-0.743***	-0.136
	(0.213)	(0.247)	(0.318)	(0.107)	(0.263)	(0.193)	(0.134)	(0.138)
Battery* Hour 18	-0.854***	-0.270	-0.617	0.066	-1.549***	-0.766**	-0.920***	-0.365**
	(0.312)	(0.321)	(0.449)	(0.141)	(0.358)	(0.302)	(0.167)	(0.185)
Battery* Hour 19	-1.453***	-0.105	-0.630	0.090	-1.125***	-0.793**	-0.922***	-0.365*
	(0.245)	(0.323)	(0.483)	(0.192)	(0.407)	(0.346)	(0.216)	(0.191)
Battery* Hour 20	-1.624***	-0.080	-0.626	0.087	-0.650*	-0.780**	-0.671***	-0.365*
	(0.282)	(0.320)	(0.546)	(0.194)	(0.360)	(0.344)	(0.209)	(0.191)
Battery* Hour 21	-0.939***	-0.067	-0.360	0.087	-0.423	-0.779**	-0.651***	-0.368*
	(0.249)	(0.317)	(0.577)	(0.194)	(0.317)	(0.344)	(0.170)	(0.190)
Battery* Hour 22	-0.207	-0.065	-0.098	0.084	-0.101	-0.778**	-0.509***	-0.370*
	(0.379)	(0.317)	(0.606)	(0.195)	(0.309)	(0.344)	(0.194)	(0.190)

Battery* Hour 23	0.644**	-0.062	-0.119	0.082	-0.046	-0.779**	-0.491**	-0.372*
	(0.305)	(0.315)	(0.456)	(0.196)	(0.316)	(0.344)	(0.215)	(0.190)
Battery* Hour 24	0.642**	-0.059	-0.111	0.082	-0.044	-0.779**	-0.486**	-0.372*
	(0.305)	(0.314)	(0.454)	(0.196)	(0.316)	(0.344)	(0.216)	(0.190)
Electricity price	-2.115***	0.402***	-6.064***	0.243	10.892***	0.710**	0.000	0.000
	(0.294)	(0.132)	(0.660)	(0.186)	(1.084)	(0.330)	(.)	(.)
Constant	1.476***	0.276***	1.758***	-0.032	-0.128	0.165***	1.050***	-0.090***
	(0.121)	(0.039)	(0.095)	(0.050)	(0.129)	(0.049)	(0.039)	(0.024)
HDD control	Yes	Yes	Yes	Yes	Yes	Yes	Yes	Yes
CDD control	Yes	Yes	Yes	Yes	Yes	Yes	Yes	Yes
Fixed effects								
Household	Yes	Yes	Yes	Yes	Yes	Yes	Yes	Yes
Day of sample	Yes	Yes	Yes	Yes	Yes	Yes	Yes	Yes
Hour of day	Yes	Yes	Yes	Yes	Yes	Yes	Yes	Yes
N	1106831	969957	1203797	1020805	4186095	3616772	4426192	3745697

Standard errors in parentheses *p<0.1 **p<0.05 ***p<0.01

Table S5 (b). Regression results for Figure 3, by rate, Rate E21 and Rate E23, PSM. Related to Figure 3.

	Rate E21				Rate E23			
	Summer, Delivered	Summer, Received	Winter, Delivered	Winter, Received	Summer, Delivered	Summer, Received	Winter, Delivered	Winter, Received
Battery* Hour 1	-0.868	0.193	-0.012	0.084	-0.215	-0.202	-0.148	-0.118
	(0.566)	(0.322)	(0.393)	(0.192)	(0.432)	(0.216)	(0.341)	(0.153)
Battery* Hour 2	-0.799	0.193	0.104	0.086	0.141	-0.200	-0.170	-0.118
	(0.530)	(0.322)	(0.442)	(0.192)	(0.385)	(0.216)	(0.208)	(0.154)
Battery* Hour 3	-0.735	0.195	0.190	0.083	0.162	-0.199	-0.242	-0.124
	(0.463)	(0.320)	(0.427)	(0.193)	(0.321)	(0.216)	(0.193)	(0.155)
Battery* Hour 4	-0.579	0.193	0.284	0.094	0.200	-0.198	-0.266	-0.113
	(0.411)	(0.320)	(0.417)	(0.191)	(0.302)	(0.216)	(0.168)	(0.154)
Battery* Hour 5	-0.703**	0.192	-0.290	0.096	0.121	-0.199	-0.454**	-0.113
	(0.318)	(0.320)	(0.267)	(0.191)	(0.304)	(0.216)	(0.188)	(0.154)
Battery* Hour 6	-0.467	0.191	-0.447	0.097	0.064	-0.199	-0.478**	-0.113
	(0.520)	(0.320)	(0.269)	(0.191)	(0.298)	(0.216)	(0.181)	(0.154)
Battery* Hour 7	-0.977**	0.097	-0.838*	0.085	0.298	-0.316	-0.221	-0.131
	(0.434)	(0.307)	(0.427)	(0.191)	(0.262)	(0.213)	(0.206)	(0.153)
Battery* Hour 8	-0.745**	-0.313	-0.519*	-0.035	0.451*	-0.810***	-0.132	-0.294**
	(0.297)	(0.314)	(0.272)	(0.187)	(0.247)	(0.228)	(0.231)	(0.129)
Battery* Hour 9	-0.353	-0.976**	-0.279	-0.366	0.527	-1.140**	0.106	-0.686***
	(0.303)	(0.382)	(0.314)	(0.239)	(0.370)	(0.423)	(0.324)	(0.211)
Battery* Hour 10	0.555	-1.367*	-0.036	-0.877*	0.545	-1.218	0.088	-1.029**
	(0.532)	(0.725)	(0.393)	(0.450)	(0.396)	(0.754)	(0.283)	(0.468)

Battery* Hour 11	0.517	-1.824*	0.060	-1.444*	0.489	-1.187	-0.000	-1.178
	(0.497)	(0.912)	(0.321)	(0.684)	(0.391)	(0.993)	(0.280)	(0.716)
Battery* Hour 12	0.626	-1.942*	-0.012	-1.657*	0.489	-0.978	-0.028	-0.968
	(0.537)	(0.927)	(0.318)	(0.784)	(0.410)	(1.136)	(0.288)	(0.874)
Battery* Hour 13	0.731	-1.504*	0.055	-1.248	0.325	-0.640	-0.027	-0.655
	(0.626)	(0.819)	(0.358)	(0.739)	(0.382)	(1.194)	(0.288)	(0.844)
Battery* Hour 14	0.842	-1.439**	0.116	-0.790	0.254	-0.334	-0.060	-0.202
	(0.684)	(0.610)	(0.380)	(0.685)	(0.390)	(1.018)	(0.280)	(0.699)
Battery* Hour 15	0.934*	-1.500***	0.176	-0.361	-0.242	-0.063	-0.128	0.152
	(0.518)	(0.231)	(0.381)	(0.595)	(0.267)	(0.790)	(0.262)	(0.516)
Battery* Hour 16	-0.229	-1.036***	-0.080	-0.144	-0.854***	-0.178	-0.349	0.159
	(0.281)	(0.185)	(0.328)	(0.365)	(0.302)	(0.440)	(0.236)	(0.292)
Battery* Hour 17	-0.297	-0.520*	-0.382	0.078	-1.274***	-0.292	-0.651***	0.040
	(0.394)	(0.263)	(0.337)	(0.119)	(0.382)	(0.173)	(0.196)	(0.144)
Battery* Hour 18	-0.406	-0.142	-0.724	0.080	-1.836***	-0.259	-1.123***	-0.089
	(0.470)	(0.332)	(0.481)	(0.144)	(0.539)	(0.201)	(0.229)	(0.153)
Battery* Hour 19	-0.891**	0.128	-0.771	0.087	-1.747***	-0.220	-1.334***	-0.105
	(0.354)	(0.328)	(0.517)	(0.184)	(0.583)	(0.223)	(0.282)	(0.150)
Battery* Hour 20	-1.179***	0.177	-0.815	0.082	-1.268**	-0.209	-1.096***	-0.111
	(0.379)	(0.326)	(0.565)	(0.187)	(0.499)	(0.221)	(0.288)	(0.150)
Battery* Hour 21	-0.952**	0.190	-0.514	0.082	-0.968**	-0.203	-0.959***	-0.116
	(0.390)	(0.324)	(0.573)	(0.187)	(0.447)	(0.218)	(0.279)	(0.150)
Battery* Hour 22	-0.480	0.191	-0.240	0.079	-0.273	-0.201	-0.643**	-0.121
	(0.587)	(0.325)	(0.612)	(0.189)	(0.506)	(0.217)	(0.303)	(0.150)

Battery* Hour 23	0.032	0.194	-0.313	0.078	-0.184	-0.201	-0.463	-0.125
	(0.478)	(0.324)	(0.517)	(0.189)	(0.464)	(0.217)	(0.305)	(0.151)
Battery* Hour 24	0.031	0.194	-0.303	0.078	-0.181	-0.201	-0.449	-0.127
	(0.478)	(0.322)	(0.516)	(0.190)	(0.464)	(0.216)	(0.305)	(0.151)
Electricity price	- 1.437***	0.294	- 8.637***	-0.293	13.137***	0.956	0.000	0.000
	(0.365)	(0.227)	(2.109)	(0.530)	(2.995)	(0.954)	(.)	(.)
Constant	2.908***	0.199	2.348***	-0.051	0.147	0.119	1.586***	-0.006
	(0.478)	(0.161)	(0.524)	(0.144)	(0.336)	(0.125)	(0.247)	(0.163)
HDD control	Yes	Yes	Yes	Yes	Yes	Yes	Yes	Yes
CDD control	Yes	Yes	Yes	Yes	Yes	Yes	Yes	Yes
Fixed effects								
Household	Yes	Yes	Yes	Yes	Yes	Yes	Yes	Yes
Day of sample	Yes	Yes	Yes	Yes	Yes	Yes	Yes	Yes
Hour of day	Yes	Yes	Yes	Yes	Yes	Yes	Yes	Yes
N	119030	101781	138282	114705	289271	238517	293389	232189

Standard errors in parentheses *p<0.1 **p<0.05 ***p<0.01

Table S6 (a). Regression results for Figure 4, by rate, Rate E26 and Rate E27, no matching. Related to Figure 4.

	Rate E26				Rate E27			
	Summer, Delivered	Summer, Received	Winter, Delivered	Winter, Received	Summer, Delivered	Summer, Received	Winter, Delivered	Winter, Received
Battery* Hour 1	-0.746*	-0.309	0.011	-0.239	0.198	-0.218	-0.047	-0.115
	(0.418)	(0.349)	(0.245)	(0.189)	(0.294)	(0.169)	(0.204)	(0.088)
Battery* Hour 2	-0.762**	-0.307	-0.164	-0.238	0.550	-0.217	0.110	-0.114
	(0.304)	(0.350)	(0.186)	(0.189)	(0.435)	(0.169)	(0.276)	(0.088)
Battery* Hour 3	-0.552***	-0.307	-0.227	-0.240	0.444	-0.217	0.058	-0.114
	(0.209)	(0.352)	(0.177)	(0.189)	(0.360)	(0.169)	(0.242)	(0.088)
Battery* Hour 4	-0.414**	-0.307	-0.258	-0.232	0.178	-0.217	-0.156	-0.111
	(0.161)	(0.352)	(0.159)	(0.189)	(0.199)	(0.169)	(0.147)	(0.088)
Battery* Hour 5	-0.257	-0.308	-0.216	-0.231	0.000	-0.218	-0.172	-0.110
	(0.225)	(0.352)	(0.167)	(0.189)	(0.202)	(0.170)	(0.174)	(0.088)
Battery* Hour 6	-0.430*	-0.309	-0.494***	-0.230	-0.076	-0.218	-0.550***	-0.104
	(0.222)	(0.352)	(0.179)	(0.188)	(0.244)	(0.170)	(0.148)	(0.089)
Battery* Hour 7	-0.797***	-0.348	-0.617***	-0.235	-0.021	-0.230	-0.509***	-0.107
	(0.282)	(0.353)	(0.207)	(0.188)	(0.217)	(0.160)	(0.161)	(0.088)
Battery* Hour 8	-0.783*	-0.633*	-0.606***	-0.344*	0.070	-0.326***	-0.221	-0.153
	(0.402)	(0.361)	(0.141)	(0.188)	(0.199)	(0.125)	(0.183)	(0.095)
Battery* Hour 9	-0.291	-1.186***	-0.377***	-0.793***	0.124	-0.537**	0.008	-0.372**
	(0.505)	(0.385)	(0.082)	(0.191)	(0.178)	(0.208)	(0.148)	(0.174)
Battery* Hour 10	-0.047	-1.760***	-0.174	-1.424***	0.227	-0.767**	0.339*	-0.926***
	(0.544)	(0.381)	(0.215)	(0.202)	(0.184)	(0.318)	(0.194)	(0.122)

Battery* Hour 11	0.013	-1.907***	-0.195	-1.851***	0.083	-0.857**	0.303*	-1.114***
	(0.510)	(0.274)	(0.270)	(0.187)	(0.184)	(0.375)	(0.177)	(0.196)
Battery* Hour 12	-0.185	-1.225***	-0.305	-1.672***	-0.012	-0.678*	0.179	-1.104***
	(0.450)	(0.210)	(0.277)	(0.309)	(0.192)	(0.380)	(0.132)	(0.249)
Battery* Hour 13	-0.643**	0.480	-0.286	-1.008*	0.064	-0.426	-0.030	-0.959***
	(0.326)	(0.912)	(0.275)	(0.568)	(0.243)	(0.370)	(0.189)	(0.252)
Battery* Hour 14	-0.756**	1.121	-0.334	-0.148	-0.103	-0.171	-0.054	-0.538**
	(0.310)	(0.993)	(0.275)	(0.639)	(0.223)	(0.359)	(0.181)	(0.230)
Battery* Hour 15	-1.125***	0.802	-0.377	0.163	-0.826***	0.018	-0.126	-0.152
	(0.243)	(0.798)	(0.266)	(0.518)	(0.221)	(0.299)	(0.181)	(0.203)
Battery* Hour 16	-1.359***	0.493	-0.441*	0.005	-1.084***	0.093	-0.226	0.039
	(0.237)	(0.543)	(0.238)	(0.286)	(0.224)	(0.206)	(0.176)	(0.151)
Battery* Hour 17	-1.649***	0.137	-0.572***	-0.126	-1.461***	-0.030	-0.325**	0.018
	(0.226)	(0.234)	(0.180)	(0.103)	(0.208)	(0.123)	(0.158)	(0.089)
Battery* Hour 18	-0.711	-0.316	-0.698***	-0.222*	-1.745***	-0.171	-0.702***	-0.049
	(0.792)	(0.212)	(0.075)	(0.133)	(0.185)	(0.122)	(0.144)	(0.087)
Battery* Hour 19	-0.205	-0.372	-0.942***	-0.242	-1.887***	-0.207	-0.861***	-0.121
	(1.063)	(0.340)	(0.200)	(0.187)	(0.169)	(0.159)	(0.111)	(0.089)
Battery* Hour 20	-0.326	-0.349	-0.954***	-0.243	-1.621***	-0.218	-0.750***	-0.120
	(0.647)	(0.345)	(0.237)	(0.189)	(0.176)	(0.168)	(0.136)	(0.088)
Battery* Hour 21	-0.221	-0.333	-0.881***	-0.245	-0.460	-0.218	-0.538**	-0.121
	(0.195)	(0.348)	(0.267)	(0.189)	(0.517)	(0.168)	(0.245)	(0.088)
Battery* Hour 22	-0.590***	-0.326	-0.461	-0.248	0.128	-0.217	0.426	-0.124
	(0.186)	(0.350)	(0.322)	(0.188)	(0.534)	(0.168)	(0.320)	(0.088)

Battery* Hour 23	-0.580	-0.321	0.004	-0.249	-0.003	-0.218	0.309	-0.124
	(0.362)	(0.350)	(0.435)	(0.188)	(0.438)	(0.169)	(0.333)	(0.088)
Battery* Hour 24	-0.585	-0.317	0.013	-0.249	-0.003	-0.218	0.314	-0.123
	(0.361)	(0.351)	(0.435)	(0.188)	(0.438)	(0.169)	(0.333)	(0.088)
Electricity price	-1.652***	0.280***	0.393	-1.365***	0.036***	-0.063***	0.028***	-0.003
	(0.398)	(0.052)	(0.607)	(0.235)	(0.010)	(0.005)	(0.010)	(0.005)
Constant	1.641***	0.233***	1.439***	0.372***	2.725***	0.221***	2.613***	0.098
	(0.085)	(0.030)	(0.077)	(0.063)	(0.116)	(0.030)	(0.237)	(0.063)
HDD control	Yes	Yes	Yes	Yes	Yes	Yes	Yes	Yes
CDD control	Yes	Yes	Yes	Yes	Yes	Yes	Yes	Yes
Fixed effects								
Household	Yes	Yes	Yes	Yes	Yes	Yes	Yes	Yes
Day of sample	Yes	Yes	Yes	Yes	Yes	Yes	Yes	Yes
Hour of day	Yes	Yes	Yes	Yes	Yes	Yes	Yes	Yes
N	1503150	1214508	1565798	1229526	1815399	1815399	2334784	2334784

Standard errors in parentheses *p<0.1 **p<0.05 ***p<0.01

Table S6 (b). Regression results for Figure 4, by rate, Rate E26 and Rate E27, PSM. Related to Figure 4.

	Rate E26				Rate E27			
	Summer, Delivered	Summer, Received	Winter, Delivered	Winter, Received	Summer, Delivered	Summer, Received	Winter, Delivered	Winter, Received
Battery* Hour 1	-1.109**	-0.155	-0.157	-0.170	-0.150	-0.193	-0.215	-0.164*
	(0.462)	(0.319)	(0.298)	(0.191)	(0.305)	(0.164)	(0.213)	(0.092)
Battery* Hour 2	-1.109***	-0.153	-0.319	-0.169	-0.110	-0.192	-0.203	-0.162*
	(0.337)	(0.321)	(0.227)	(0.191)	(0.266)	(0.164)	(0.201)	(0.093)
Battery* Hour 3	-0.874***	-0.154	-0.338	-0.175	-0.075	-0.192	-0.154	-0.164*
	(0.270)	(0.323)	(0.217)	(0.193)	(0.242)	(0.164)	(0.194)	(0.093)
Battery* Hour 4	-0.741***	-0.156	-0.363*	-0.163	-0.077	-0.193	-0.247	-0.158*
	(0.253)	(0.324)	(0.207)	(0.191)	(0.230)	(0.165)	(0.169)	(0.092)
Battery* Hour 5	-0.462	-0.157	-0.217	-0.163	-0.118	-0.194	-0.200	-0.156*
	(0.293)	(0.324)	(0.198)	(0.192)	(0.217)	(0.165)	(0.183)	(0.093)
Battery* Hour 6	-0.511**	-0.158	-0.455**	-0.161	-0.052	-0.195	-0.382**	-0.149
	(0.241)	(0.325)	(0.189)	(0.192)	(0.263)	(0.165)	(0.167)	(0.094)
Battery* Hour 7	-0.767**	-0.192	-0.615**	-0.164	-0.016	-0.193	-0.339*	-0.150
	(0.301)	(0.328)	(0.247)	(0.193)	(0.245)	(0.154)	(0.187)	(0.094)
Battery* Hour 8	-0.562	-0.594	-0.492**	-0.281	-0.007	-0.213*	-0.087	-0.168
	(0.405)	(0.365)	(0.201)	(0.188)	(0.235)	(0.119)	(0.187)	(0.101)
Battery* Hour 9	0.003	-1.296**	-0.174	-0.809***	0.047	-0.308	0.100	-0.311
	(0.492)	(0.462)	(0.145)	(0.207)	(0.245)	(0.234)	(0.167)	(0.189)
Battery* Hour 10	0.326	-1.920***	0.117	-1.550***	0.181	-0.548	0.391*	-0.856***
	(0.523)	(0.531)	(0.225)	(0.317)	(0.282)	(0.365)	(0.231)	(0.183)

Battery* Hour 11	0.459	-2.102***	0.115	-1.980***	0.202	-0.771*	0.295	-1.038***
	(0.496)	(0.481)	(0.263)	(0.382)	(0.267)	(0.430)	(0.226)	(0.263)
Battery* Hour 12	0.332	-1.483***	0.005	-1.935***	0.191	-0.699	0.177	-1.051***
	(0.435)	(0.310)	(0.264)	(0.415)	(0.269)	(0.433)	(0.182)	(0.312)
Battery* Hour 13	-0.090	0.205	0.002	-1.316**	0.250	-0.498	-0.035	-0.905***
	(0.318)	(0.834)	(0.249)	(0.591)	(0.337)	(0.414)	(0.192)	(0.313)
Battery* Hour 14	-0.341	0.881	-0.049	-0.445	0.067	-0.298	-0.066	-0.511*
	(0.310)	(0.936)	(0.247)	(0.640)	(0.334)	(0.396)	(0.191)	(0.270)
Battery* Hour 15	-0.851***	0.632	-0.129	-0.015	-0.600*	-0.102	-0.149	-0.161
	(0.250)	(0.805)	(0.241)	(0.534)	(0.331)	(0.333)	(0.190)	(0.219)
Battery* Hour 16	-1.140***	0.446	-0.203	-0.085	-0.874**	-0.019	-0.258	-0.003
	(0.254)	(0.616)	(0.228)	(0.313)	(0.378)	(0.232)	(0.220)	(0.154)
Battery* Hour 17	-1.620***	0.178	-0.421*	-0.142	-1.303***	-0.124	-0.434*	-0.022
	(0.294)	(0.317)	(0.219)	(0.118)	(0.396)	(0.154)	(0.242)	(0.101)
Battery* Hour 18	-1.034	-0.182	-0.755***	-0.144	-1.607***	-0.267*	-0.790***	-0.088
	(0.863)	(0.190)	(0.176)	(0.124)	(0.333)	(0.148)	(0.216)	(0.094)
Battery* Hour 19	-0.617	-0.203	-1.100***	-0.152	-1.885***	-0.218	-0.961***	-0.157*
	(1.146)	(0.310)	(0.198)	(0.177)	(0.284)	(0.156)	(0.171)	(0.091)
Battery* Hour 20	-0.732	-0.186	-1.165***	-0.159	-1.672***	-0.195	-0.876***	-0.160*
	(0.741)	(0.315)	(0.208)	(0.180)	(0.263)	(0.163)	(0.169)	(0.091)
Battery* Hour 21	-0.495	-0.174	-1.090***	-0.163	-0.949	-0.193	-0.780***	-0.163*
	(0.388)	(0.318)	(0.255)	(0.181)	(0.586)	(0.163)	(0.272)	(0.091)
Battery* Hour 22	-0.907**	-0.169	-0.596*	-0.166	-0.312	-0.191	0.270	-0.166*

	(0.334)	(0.320)	(0.300)	(0.181)	(0.550)	(0.163)	(0.345)	(0.091)
Battery* Hour 23	-1.001**	-0.165	-0.153	-0.169	-0.683	-0.192	-0.014	-0.167*
	(0.462)	(0.320)	(0.421)	(0.182)	(0.461)	(0.164)	(0.339)	(0.091)
Battery* Hour 24	-1.002**	-0.162	-0.146	-0.169	-0.684	-0.193	-0.008	-0.166*
	(0.463)	(0.322)	(0.421)	(0.183)	(0.460)	(0.164)	(0.339)	(0.092)
Electricity price	-0.377	0.370	2.353	- 2.563***	- 36.739***	3.063**	- 71.139***	-1.993
	(0.677)	(0.257)	(1.860)	(0.562)	(5.839)	(1.475)	(18.113)	(4.452)
Constant	1.528***	0.262*	1.506***	0.377	3.572***	0.158*	4.307***	0.012
	(0.260)	(0.138)	(0.285)	(0.224)	(0.365)	(0.079)	(0.726)	(0.198)
HDD control	Yes	Yes	Yes	Yes	Yes	Yes	Yes	Yes
CDD control	Yes	Yes	Yes	Yes	Yes	Yes	Yes	Yes
Fixed effects								
Household	Yes	Yes	Yes	Yes	Yes	Yes	Yes	Yes
Day of sample	Yes	Yes	Yes	Yes	Yes	Yes	Yes	Yes
Hour of day	Yes	Yes	Yes	Yes	Yes	Yes	Yes	Yes
N	138277	111197	145154	115239	271571	271571	319784	319784

Standard errors in parentheses *p<0.1 **p<0.05 ***p<0.01

Table S7. Regression results for Figure S1, no control consumers and group-specific time trend. Related to STAR Methods.

	No control				Time trend			
	Summer, Delivered	Summer, Received	Winter, Delivered	Winter, Received	Summer, Delivered	Summer, Received	Winter, Delivered	Winter, Received
Battery* Hour 1	-0.200	0.074***	-0.302*	-0.651***	0.441**	-0.209	0.187	-0.063
	(0.246)	(0.015)	(0.178)	(0.047)	(0.209)	(0.155)	(0.150)	(0.114)
Battery* Hour 2	0.030	0.087***	-0.189	-0.650***	0.541**	-0.208	0.161	-0.061
	(0.246)	(0.017)	(0.185)	(0.047)	(0.233)	(0.155)	(0.162)	(0.114)
Battery* Hour 3	0.078	0.096***	-0.179	-0.649***	0.453**	-0.206	0.071	-0.064
	(0.231)	(0.019)	(0.175)	(0.048)	(0.218)	(0.155)	(0.154)	(0.114)
Battery* Hour 4	0.100	0.104***	-0.124	-0.649***	0.318	-0.205	-0.020	-0.059
	(0.217)	(0.021)	(0.170)	(0.048)	(0.209)	(0.155)	(0.155)	(0.114)
Battery* Hour 5	-0.046	0.112***	-0.213	-0.649***	0.093	-0.205	-0.186	-0.058
	(0.186)	(0.022)	(0.140)	(0.048)	(0.185)	(0.155)	(0.115)	(0.115)
Battery* Hour 6	-0.251*	0.126***	-0.468***	-0.649***	-0.211	-0.206	-0.590***	-0.054
	(0.133)	(0.023)	(0.126)	(0.049)	(0.153)	(0.155)	(0.122)	(0.115)
Battery* Hour 7	-0.133	0.086**	-0.394***	-0.648***	-0.311**	-0.246	-0.622***	-0.062
	(0.142)	(0.033)	(0.126)	(0.049)	(0.154)	(0.152)	(0.143)	(0.115)
Battery* Hour 8	-0.140	-0.169	-0.310***	-0.567***	-0.299**	-0.438***	-0.500***	-0.149
	(0.144)	(0.113)	(0.102)	(0.078)	(0.138)	(0.110)	(0.106)	(0.106)
Battery* Hour 9	-0.098	1.208***	-0.186*	-0.307	-0.199	-0.731***	-0.288***	-0.476***
	(0.158)	(0.212)	(0.097)	(0.319)	(0.161)	(0.158)	(0.098)	(0.104)
Battery* Hour 10	0.095	3.306***	0.038	0.469	-0.039	-0.966***	-0.028	-1.037***
	(0.179)	(0.245)	(0.110)	(0.835)	(0.193)	(0.296)	(0.129)	(0.127)

Battery* Hour 11	0.098	4.489***	-0.009	1.470	-0.109	-1.013***	-0.089	-1.294***
	(0.171)	(0.277)	(0.126)	(1.030)	(0.178)	(0.366)	(0.140)	(0.218)
Battery* Hour 12	0.074	4.314***	-0.064	3.152***	-0.185	-0.681*	-0.187	-1.168***
	(0.171)	(0.333)	(0.117)	(0.234)	(0.182)	(0.398)	(0.121)	(0.288)
Battery* Hour 13	0.030	3.613***	-0.113	3.522***	-0.232	-0.232	-0.230*	-0.834***
	(0.190)	(0.401)	(0.110)	(0.603)	(0.199)	(0.399)	(0.123)	(0.300)
Battery* Hour 14	-0.071	3.667***	-0.144	3.209***	-0.351*	0.021	-0.262**	-0.336
	(0.185)	(0.337)	(0.108)	(0.407)	(0.193)	(0.359)	(0.120)	(0.281)
Battery* Hour 15	-0.437***	3.203***	-0.196*	2.627***	-0.884***	0.120	-0.336***	-0.047
	(0.164)	(0.336)	(0.110)	(0.397)	(0.166)	(0.275)	(0.123)	(0.209)
Battery* Hour 16	-0.771***	0.869***	-0.245**	1.023*	-1.251***	0.084	-0.418***	0.027
	(0.152)	(0.292)	(0.099)	(0.557)	(0.125)	(0.159)	(0.099)	(0.105)
Battery* Hour 17	-1.124***	-0.258	-0.389***	0.180	-1.581***	-0.104	-0.511***	0.006
	(0.207)	(0.184)	(0.115)	(0.460)	(0.160)	(0.099)	(0.096)	(0.071)
Battery* Hour 18	-1.368***	-0.334***	-0.754***	-0.436*	-1.714***	-0.220	-0.864***	-0.024
	(0.284)	(0.090)	(0.188)	(0.253)	(0.272)	(0.136)	(0.160)	(0.109)
Battery* Hour 19	-1.427***	-0.098***	-0.896***	-0.650***	-1.679***	-0.219	-1.001***	-0.045
	(0.346)	(0.024)	(0.243)	(0.050)	(0.365)	(0.155)	(0.221)	(0.115)
Battery* Hour 20	-1.204***	-0.025***	-0.762***	-0.659***	-1.484***	-0.217	-0.918***	-0.047
	(0.349)	(0.007)	(0.254)	(0.048)	(0.369)	(0.155)	(0.242)	(0.115)
Battery* Hour 21	-0.692**	0.000	-0.678***	-0.658***	-0.530**	-0.213	-0.783***	-0.051
	(0.268)	(.)	(0.223)	(0.047)	(0.256)	(0.155)	(0.219)	(0.115)
Battery* Hour 22	-0.473**	0.027***	-0.139	-0.657***	-0.201	-0.210	-0.006	-0.055
	(0.226)	(0.006)	(0.202)	(0.046)	(0.238)	(0.156)	(0.227)	(0.115)

Battery* Hour 23	-0.722***	0.046***	-0.265	-0.654***	-0.285	-0.210	-0.019	-0.057
	(0.233)	(0.010)	(0.181)	(0.046)	(0.244)	(0.155)	(0.223)	(0.115)
Battery* Hour 24	-0.718***	0.061***	-0.259	-0.653***	-0.284	-0.209	-0.013	-0.058
	(0.234)	(0.013)	(0.182)	(0.046)	(0.244)	(0.155)	(0.224)	(0.114)
Electricity price	-1.739*	0.000	-2.110	-2.588	-1.409***	0.296**	-1.674**	-0.021
	(0.939)	(.)	(3.074)	(13.404)	(0.250)	(0.120)	(0.733)	(0.452)
Constant	2.025***	0.297**	2.117***	0.098	1.409***	0.334***	1.403	-0.004
	(0.208)	(0.121)	(0.294)	(1.024)	(0.042)	(0.024)	(1.332)	(0.040)
HDD control	Yes	Yes	Yes	Yes	Yes	Yes	Yes	Yes
CDD control	Yes	Yes	Yes	Yes	Yes	Yes	Yes	Yes
Fixed effects								
Household	Yes	Yes	Yes	Yes	Yes	Yes	Yes	Yes
Day of sample	Yes	Yes	Yes	Yes	Yes	Yes	Yes	Yes
Hour of day	Yes	Yes	Yes	Yes	Yes	Yes	Yes	Yes
N	959364	133547	1036920	215751	13411814	6474670	14539741	8417887

Standard errors in parentheses *p<0.1 **p<0.05 ***p<0.01
